# High Levels of Structural Diversity Observed in Microcystins from *Microcystis* CAWBG11 and Characterization of Six New Microcystin Congeners

**DOI:** 10.3390/md12115372

**Published:** 2014-11-13

**Authors:** Jonathan Puddick, Michèle R. Prinsep, Susanna A. Wood, Sangata A. F. Kaufononga, Stephen Craig Cary, David P. Hamilton

**Affiliations:** 1 Cawthron Institute, Private Bag 2, Nelson 7010, New Zealand; E-Mail: susie.wood@cawthron.org.nz; 2 Chemistry Department, School of Science, University of Waikato, Private Bag 3105, Hamilton 3240, New Zealand; E-Mails: m.prinsep@waikato.ac.nz (M.R.P.); sfk1@students.waikato.ac.nz (S.A.F.K.); 3 Biology Department, School of Science, University of Waikato, Private Bag 3105, Hamilton 3240, New Zealand; E-Mail: caryc@waikato.ac.nz; 4 Environmental Research Institute, University of Waikato, Private Bag 3105, Hamilton 3240, New Zealand; E-Mail: d.hamilton@waikato.ac.nz

**Keywords:** microcystins, *Microcystis* CAWBG11, microcystin diversity, mass spectrometry

## Abstract

Microcystins (MCs) are cyclic peptides produced by cyanobacteria, which can be harmful to humans and animals when ingested. Differences in the coding of the non-ribosomal peptide synthetase/polyketide synthase enzyme complex responsible for microcystin production have resulted in more than 100 microcystin variants being reported to date. The microcystin diversity of *Microcystis* CAWBG11 was investigated using matrix-assisted laser desorption/ionization-time of flight mass spectrometry and liquid chromatography-mass spectrometry. This revealed that CAWBG11 simultaneously produced 21 known microcystins and six new congeners: [Asp^3^] MC-RA, [Asp^3^] MC-RAba, [Asp^3^] MC-FA, [Asp^3^] MC-WA, MC-FAba and MC-FL. The new congeners were putatively characterized by tandem mass spectrometry and chemical derivatization. A survey of the microcystin congeners produced by 49 cyanobacterial strains documented in scientific literature showed that cyanobacteria generally produce four microcystin congeners, but strains which produce up to 47 microcystin congeners have been reported. *Microcystis* CAWBG11 (which produces at least 27 congeners) was positioned in the top ten percentile of the strains surveyed, and showed fluidity of the amino acids incorporated into both position two and position four.

## 1. Introduction

Cyanobacteria (blue-green algae) are a group of ancient prokaryotic organisms which use chlorophyll-*a* to harness energy from the sun with water as the reductant [1]. Cyanobacteria primarily use carbon dioxide as a carbon source, making their need for nutrients minimal and consequently they have been reported in a wide range of environments, including extreme habitats such as geothermal springs, desert soils and Polar regions [2]. Anthropogenic eutrophication of lakes, ponds and oceans, has increased the nutrient composition of these environments and created conditions favorable for the rapid growth of some cyanobacterial species (causing blooms). The cyanobacteria comprising these blooms can produce foul tastes and odors [3], but they can also synthesize toxic secondary metabolites which are poisonous to humans and animals upon ingestion [4,5].

**Figure 1:**
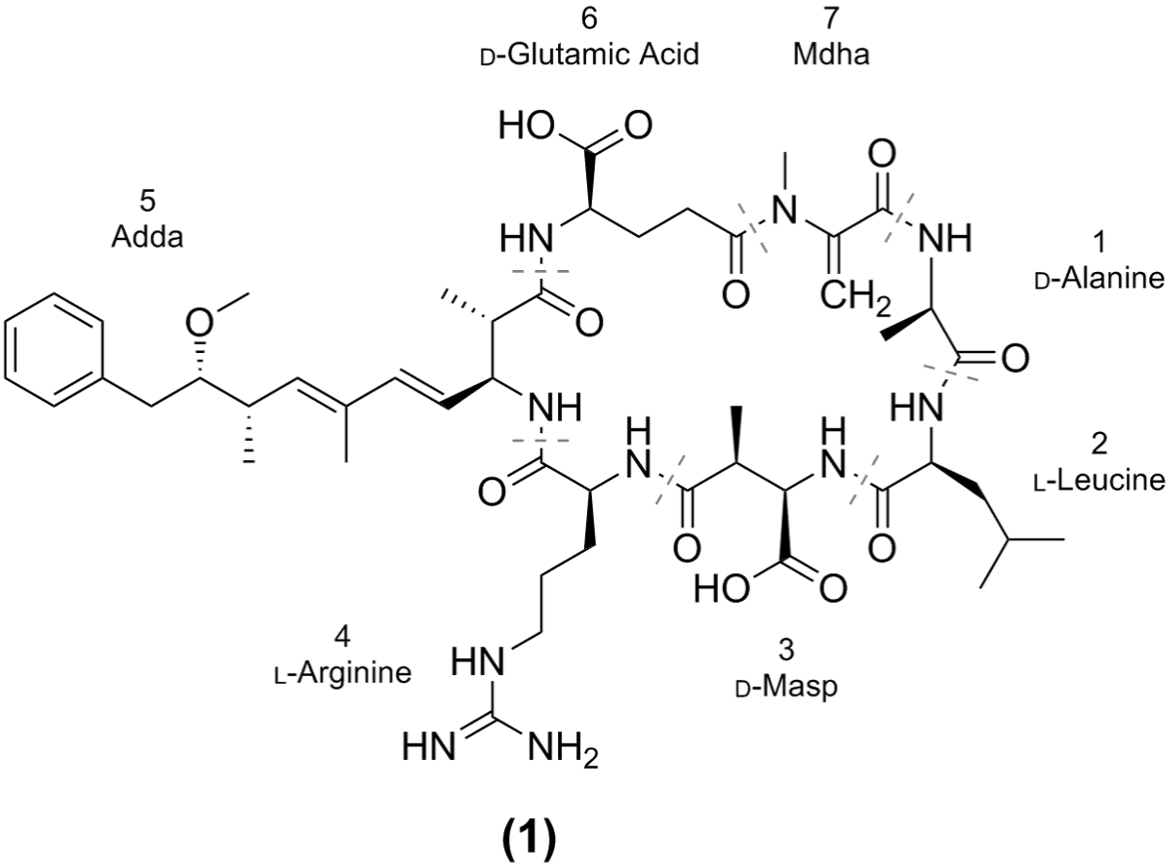
Structure of a common microcystin congener: MC-LR, where Masp is methylaspartic acid, Adda is 3-amino-9-methoxy-2,6,8-trimethyl-10-phenyldeca-4,6-dienoic acid and Mdha is *N*-methyldehydroalanine.

The most notable of these toxins are the microcystins (MCs) on account of their common production by bloom-forming cyanobacteria and their high toxicity. The microcystins are a family of cyclic heptapeptides produced via microcystin synthase, a combination non-ribosomal peptide synthetase/polyketide synthase (NRPS/PKS). As is evident in the most common variant, MC-LR (**1**; Figure 1), microcystins are generally composed of the unique β-amino acid Adda (3*S*-amino-9*S*-methoxy-2*S*,6,8*S*-trimethyl-10-phenyldeca-4*E*,6*E*-dienoic acid), d-glutamic acid (Glu), *N*-methyl dehydroalanine (Mdha), d-alanine (Ala), d-erythro-β-methylaspartic acid (Masp) and two variable l-amino acids. To date, there have been at least 100 different microcystin congeners characterized [6], mostly due to substitutions of the variable l-amino acids in positions two and four, although modifications have been reported for all of the amino acids [7].

Microcystins are produced by numerous cyanobacterial genera when the necessary microcystin synthase genes are present. These NRPS and PKS genes produce a multi-enzyme complex which sequentially adds components from malonyl-CoA, *S*-adenosyl-l-methionine and amino acids onto phenylacetate and transforms them to produce a modified peptide chain, which is condensed to form the cyclic microcystin [8,9,10,11]. The occurrence of different microcystin congeners is due to variability in the coding of the microcystin synthase genes among cyanobacterial strains. For example, at times the McyA1 *N*-methyltransferase domain can be inactive, which results in position seven desmethyl variants [10]. Furthermore, the amino acid specificity of the numerous adenylation domains is dependent upon their genetic coding and changes to this sequence can cause the domains to be specific for different amino acids or to recognize more than one amino acid [12]. Between cyanobacterial species, the substrate specificity for many of the adenylation domains is relatively conserved (positions one, three, six and seven), whilst the substrate specificity of the position two and four adenylation domains is quite varied, with a selection of amino acids being incorporated. A single strain of cyanobacteria can also produce more than one microcystin congener, which is mainly due to at least one adenylation domain possessing relaxed substrate specificity and being able to incorporate different amino acids into the structure [12,13]. Cyanobacterial strains generally produce around four microcystin congeners, in most cases: MC-LR, MC-RR and desmethyl analogues of each (Supplementary Information Table S1), and although uncommon, cyanobacterial strains which produce up to 47 microcystin congeners have been reported in the literature [14].

The different microcystin congeners range in toxicity according to their cellular availability [15] and their ability to inhibit the important eukaryotic regulatory enzymes, serine/threonine protein phosphatases 1 and 2A [16]. Microcystins predominantly affect the liver cells of mammals as they are not able to translocate the membranes of most tissues but are actively transported into the hepatocytes [17]. Inhibition of the protein phosphatases in these cells then results in excessive signaling which can lead to cellular disruption [18] or cell proliferation and tumor promotion [19]. The different microcystin congeners range in toxicity from non-toxic (e.g., [(6*Z*)-Adda^5^] MC-LR, LD_50_ > 1200 µg/kg) to highly toxic (e.g., MC-LR, LD_50_ = 50 µg/kg) by mouse bioassay [7]. Despite their toxicity, microcystins have been proposed as drug candidates for cancer treatment, due to the preferential expression of organic anion transporting polypeptides (OATPs, which actively transport microcystins into mammalian cells) in a number of cancer tissues [20]. In line with this treatment strategy, a recent study showed that certain microcystin congeners were more cytotoxic to OATP1B3-specific cell lines than OATP1B1-specific cells [21], OATP1B3 being expressed at a higher level in certain types of tumors.

A *Microcystis* species (CAWBG11) isolated from Lake Hakanoa (Huntly, New Zealand) in 2005 [22] was investigated as it produced a large number of oligopeptides including 27 microcystin congeners, six of which were new variants. Whilst cyanobacterial strains have been noted to produce more microcystin congeners [14], the diversity of microcystin variants produced by *Microcystis* CAWBG11 is not commonly observed.

## 2. Results and Discussion

### 2.1. Oligopeptide Diversity of Microcystis CAWBG11

**Figure 2:**
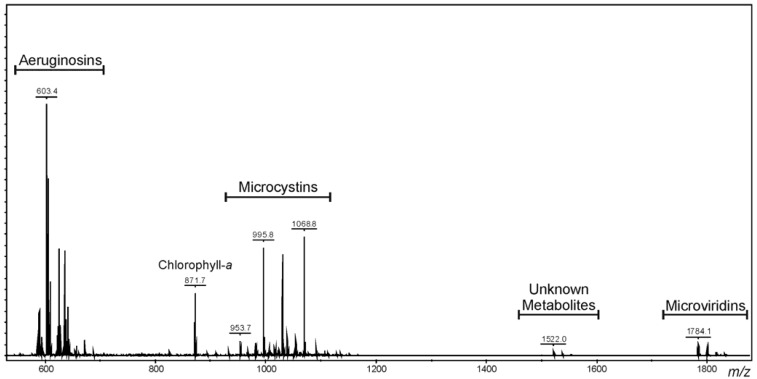
Positive ion matrix-assisted laser desorption/ionization-time of flight mass spectrum of a methanol extract of *Microcystis* CAWBG11.

A methanol extract of *Microcystis* CAWBG11 was analyzed by matrix-assisted laser desorption/ionization-time of flight mass spectrometry (MALDI-TOF MS). The resulting spectrum contained multiple ions within the 600–1800 Da mass range (Figure 2). Further investigation of these ions by MALDI post source decay (PSD) indicated the presence of linear, cyclic and tricyclic oligopeptides, including aeruginosins, microcystins, microviridins and a group of unknown metabolites with molecular weights of *ca.* 1500 Da.

The aeruginosins produced by *Microcystis* CAWBG11 included the known aeruginosins 298B [23], EI461B [24], 602 [25] and 289A [26], as well as several new aeruginosin analogues with masses of 602, 618 and 656 Da. All of the microviridins present were new variants with masses of 1760, 1764, 1778 and 1784 Da. As yet, there is little information on the unknown metabolites with molecular weights of ca. 1500 Da but characterization of new compounds from CAWBG11 forms part of the current research in our group.

**Figure 3:**
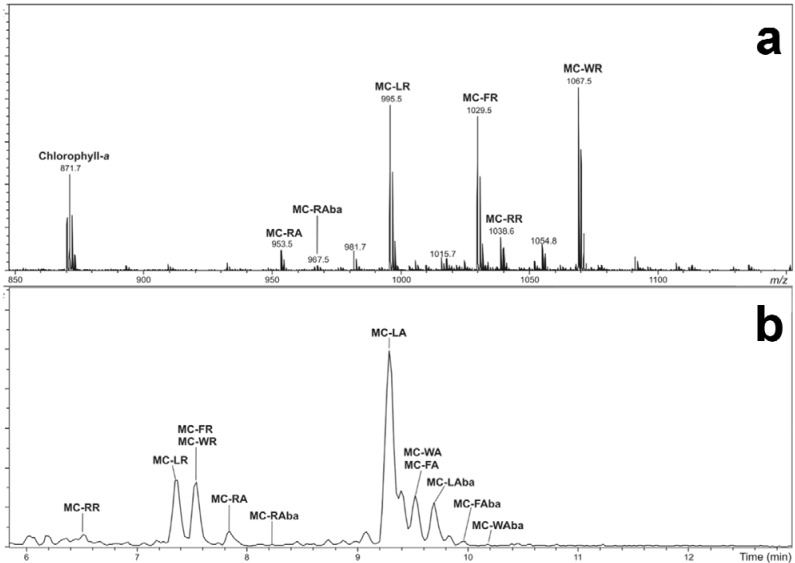
Positive ion matrix-assisted laser desorption/ionization-time of flight mass spectrum of the microcystin-containing region (*m/z* 850–1150) (**a**) and a liquid chromatography-mass spectrometry basepeak chromatogram (negative ion; *m/z* 850–1150) (**b**) of a methanol extract of *Microcystis* CAWBG11.

Also apparent in the MALDI-TOF mass spectrum was a plethora of arginine-containing microcystins (Figure 3a). Since microcystins which do not contain an arginine residue have a low ionization efficiency using MALDI [27], the microcystin diversity of *Microcystis* CAWBG11 was also investigated using high-performance liquid chromatography (HPLC) with electrospray ionization (ESI) MS. The LC-MS analysis (Figure 3b) revealed the presence of 15 microcystin congeners which were not evident from the MALDI-TOF MS analysis. In total, 27 microcystin congeners were detected (**1**–**27**; Table 1), six of which were new (**10**, **12**, **18**, **20**, **23** and **26**). The microcystins ranged in polarity from hydrophilic (MC-RR; **3**) to slightly hydrophobic (MC-WL; **27**) and in size from 895.5 Da ([Asp^3^] MC-LA; **16**) to 1067.5 Da (MC-WR; **9**). *Microcystis* CAWBG11 also contained oxidized tryptophan microcystin congeners [28] which were most likely oxidation products of the tryptophan-containing congeners present in CAWBG11. As these microcystins do not appear to be synthesized by microcystin synthase, they have not been included in this study on the microcystin diversity of *Microcystis* CAWBG11.

**Table 1:** Structures, molecular masses and retention times of the microcystins (MCs) identified in *Microcystis* CAWBG11. 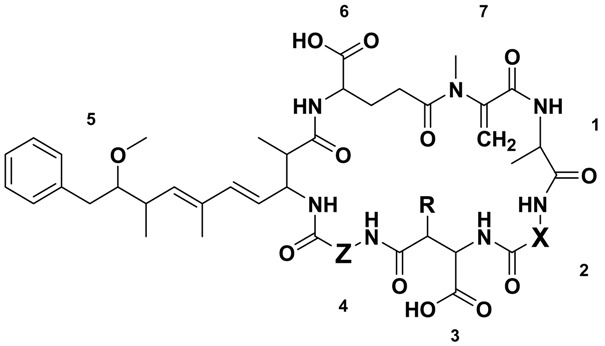

Microcystin		M*_r_^a^* (Da)	RT *^b^* (min)	X	Z	R	Reference(s)
MC-RRs	[Asp^3^] MC-RR (**2**)	1023.6	6.32	Arg	Arg	H	[29]
	MC-RR (**3**)	1037.6	6.45	Arg	Arg	CH_3_	[30,31,32]
MC-XRs	MC-YR (**4**)	1044.5	7.20	Tyr	Arg	CH_3_	[33]
	[Asp^3^] MC-LR (**5**)	980.5	7.32	Leu	Arg	H	[34,35]
	MC-LR (**1**)	994.5	7.35	Leu	Arg	CH_3_	[16,33]
	[Asp^3^] MC-FR (**6**)	1014.5	7.52	Phe	Arg	H	[36]
	MC-FR (**7**)	1028.5	7.54	Phe	Arg	CH_3_	[37]
	[Asp^3^] MC-WR (**8**)	1053.5	7.56	Trp	Arg	H	[36]
	MC-WR (**9**)	1067.5	7.60	Trp	Arg	CH_3_	[37]
MC-RZs	[Asp^3^] MC-RA (**10**)	938.5	7.80	Arg	Ala	H	This Study
	MC-RA (**11**)	952.5	7.84	Arg	Ala	CH_3_	[38,39]
	[Asp^3^] MC-RAba (**12**)	952.5	8.16	Arg	Aba	H	This Study
	MC-RAba (**13**)	966.5	8.18	Arg	Aba	CH_3_	[39]
	MC-RL (**14**)	994.5	8.35	Arg	Leu	CH_3_	[39]
MC-XAs	MC-YA (**15**)	959.5	9.01	Tyr	Ala	CH_3_	[33]
	[Asp^3^] MC-LA (**16**)	895.5	9.12	Leu	Ala	H	[40]
	MC-LA (**17**)	909.5	9.29	Leu	Ala	CH_3_	[41]
	[Asp^3^] MC-FA (**18**)	929.5	9.46	Phe	Ala	H	This Study
	MC-FA (**19**)	943.5	9.52	Phe	Ala	CH_3_	[42]
	[Asp^3^] MC-WA (**20**)	968.5	9.48	Trp	Ala	H	This Study
	MC-WA (**21**)	982.5	9.54	Trp	Ala	CH_3_	[42]
MC-XAbas	MC-LAba (**22**)	923.5	9.69	Leu	Aba	CH_3_	[40,43,44]
	MC-FAba (**23**)	957.5	9.97	Phe	Aba	CH_3_	This Study
	MC-WAba (**24**)	996.5	10.24	Trp	Aba	CH_3_	[28]
MC-XLs	MC-LL (**25**)	951.5	10.50	Leu	Leu	CH_3_	[45]
	MC-FL (**26**)	985.5	10.71	Phe	Leu	CH_3_	This Study
	MC-WL (**27**)	1024.5	10.77	Trp	Leu	CH_3_	[28]

*^a^* Molecular masses are rounded to one decimal place; *^b^* RT = Retention time on a C_18_ HPLC column as per Section 3.4.

### 2.2. Characterization of Microcystins from Microcystis CAWBG11

In order to characterize the microcystins produced by *Microcystis* CAWBG11, multiple methods of analysis were undertaken including a recently described thiol derivatization technique, tandem MS (MS/MS), high-resolution MS (HRMS) and amino acid analysis (Advanced Marfey’s method). However, many of the microcystins were present in low quantities allowing only analysis by MS/MS and thiol derivatization, including each of the new congeners reported here (**10**, **12**, **18**, **20**, **23** and **26**).

The position seven amino acid in microcystins is frequently Mdha, although the isometric amino acid, dehydrobutyrine (Dhb), has also been reported on several occasions [46,47,48]. Previously, NMR analysis of purified material (~1 mg) has been required to confirm the identity of this amino acid in microcystins. However, a recently described thiol derivatization technique has been shown to be effective for the discrimination of these two moieties [49]. When using this technique, a microcystin containing a terminal alkene, as is found in Mdha and dehydroalanine (Dha), will readily react with β-mercaptoethanol under alkaline conditions [39,50]. When Dhb is present, this reaction rate is at least two orders of magnitude slower [49]. A β-mercaptoethanol derivatization of the *Microcystis* CAWBG11 extract underwent rapid reaction (*t*_½_ < 18 min) and all of the congeners were fully derivatized within four hours indicating that all microcystins present contained either Dha or Mdha. Tandem MS analysis of each congener showed that an 83 Da moiety was present at position seven (Supplementary Information Tables S2–S7); therefore, each congener produced by CAWBG11 was designated as containing Mdha.

Many of the microcystins identified in *Microcystis* CAWBG11 have been reported previously (see Table 1) and therefore an in-depth description of their characterization is not presented here. However, tables of MS/MS fragment assignments used to confirm the structure of each microcystin detected in CAWBG11 are available in Supplementary Information Tables S2–S7.

The MC-RR microcystins (**2**–**3**) were predominantly observed as [M+2H]^2+^ ions by ESI MS, although low intensity signals for the singly-protonated ions were also observed by MALDI MS. The resulting ESI MS/MS fragment ions were a combination of singly- and doubly-charged ions (Supplementary Information Table S2). During the MS/MS analysis of the -RR congeners, doubly-protonated fragment ions were distinguished from singly-protonated ions by assessing the isotopic peak pattern. When a singly-protonated ion was present there was a difference of +1 *m/z* between the isotopic peaks and a difference of +0.5 *m/z* was observed when the ion was doubly-protonated.

The -XR and -RZ microcystins yielded predominantly [M+H]^+^ ions by both MALDI MS and ESI MS (although there were also sodium- and potassium-adducts present in the ESI mass spectra). The -XR microcystins were characterized using a combination of the MALDI PSD and ESI CID fragment ions (Supplementary Information Table S3) where the MALDI PSD spectra provided lower mass fragment ions and the ESI CID spectra provided higher mass fragments. These fragments included commonly observed elements such as a fragment of the Adda sidechain (*m/z* 135), the Adda′-Glu-Mdha fragment ion (*m/z* 375) and the Arg-Adda-Glu or Masp-Arg-Adda fragments (*m/z* 599) [43,51]. The -RZ microcystins were also characterized using both MALDI PSD and ESI CID data (Supplementary Information Table S4). Since the position of the arginine residue in the structure is inverted in the -RZ microcystins (and the guanidinium group provides the major ionization point), several of the MS/MS fragment ions observed were different from those seen in the -XR congeners. For example, while the *m/z* 599 ion (Arg-Adda-Glu or Masp-Arg-Adda) [51] is one of the major fragment ions in a -XR microcystin congener, a *m/z* 440 fragment ion (Glu-Mdha-Ala-Arg and Mdha-Ala-Arg-Masp) was present in the -RZ congeners [52].

The -XA, -XAba and -XL microcystins did not ionize efficiently by MALDI MS and yielded predominantly [M+Na]^+^ and [M+K]^+^ adduct ions by ESI MS. However, there were low levels of [M+H]^+^ ions present which were used for the MS/MS characterization. The -XA microcystins (Supplementary Information Table S5), -XAba microcystins (Supplementary Information Table S6) and -XL microcystins (Supplementary Information Table S7) produced many of the fragment ions observed for the other microcystin congeners analyzed but also possessed an additional ammonia-loss fragment ion series which started from Adda-Glu-Mdha minus NH_3_ (*m/z* 509). The -XL microcystins produced several intense fragment ions that could not be assigned during the course of this study, for example, an *m/z* 535 was present in MS/MS spectra of these congeners (Supplementary Information Table S7). As this fragment had the same *m/z* in each of the -XL microcystin congeners (**25**–**27**), it is most likely from a common portion of the structure. The mass of the other unassigned fragment ion differed in each of the microcystins: *m/z* 440 in MC-LL (**25**), *m/z* 474 in MC-FL (**26**) and *m/z* 513 in MC-WL (**27**). The mass increments between each of these fragments were the same as those between leucine, phenylalanine and tryptophan and are therefore most likely to result from a portion of the structure which contains the position two amino acid.

Semi-pure mixtures of the microcystins were analyzed by HRMS to determine accurate masses and confirm the proposed molecular formulae of the compounds. The CAWBG11 microcystins which were present in sufficient quantities to be detected using the HRESIMS instrumentation available yielded accurate masses which were consistent with their proposed structures (±5 ppm; Supplementary Information Table S8). The accurate mass for MC-RR was determined from the doubly-protonated ion, the -XR and -RZ congeners were determined from the singly-protonated ions, whilst the -XA and -XAba congeners were determined using the sodium adduct ions.

Several of the microcystins in CAWBG11 were present in sufficient quantities to isolate and perform NMR and amino acid analysis to further confirm their structures and determine the stereochemistry of certain structural elements. The full characterization of MC-FA (**19**) and MC-WA (**21**) from *Microcystis* CAWBG11 was recently reported [42] and determined that these microcystins contained d-Ala, d-Glu, d-Masp, Mdha, 3*S*,4*E*,6*E*-Adda and two l-amino acids. Hydrolyzates of purified MC-RA (**11**) and MC-RAba (**12**) from *Microcystis* CAWBG11 were also subjected to Advanced Marfey’s amino acid analysis which yielded complimentary results (Supplementary Information Figures S1–S2): that the microcystins contained d-Glu, d-Masp, d-Ala, 3*S*-Adda, two l-amino acids and *N*-methylamine (which is indicative of Mdha [53]). It also confirmed that the MC-RAba found in *Microcystis* CAWBG11 contained l-2-aminobutanoic acid (Aba) and not the isomeric version (2-amino-*iso*-butanoic acid; Aib). This was consistent with the only other study of an Aba-containing microcystin, where differentiation was made between Aba and Aib [54]. It was assumed that the other Aba-containing microcystins in CAWBG11 also contain this amino acid variant.

### 2.3. Tandem Mass Spectrometry Characterization of New Microcystins from Microcystis CAWBG11

**Figure 4:**
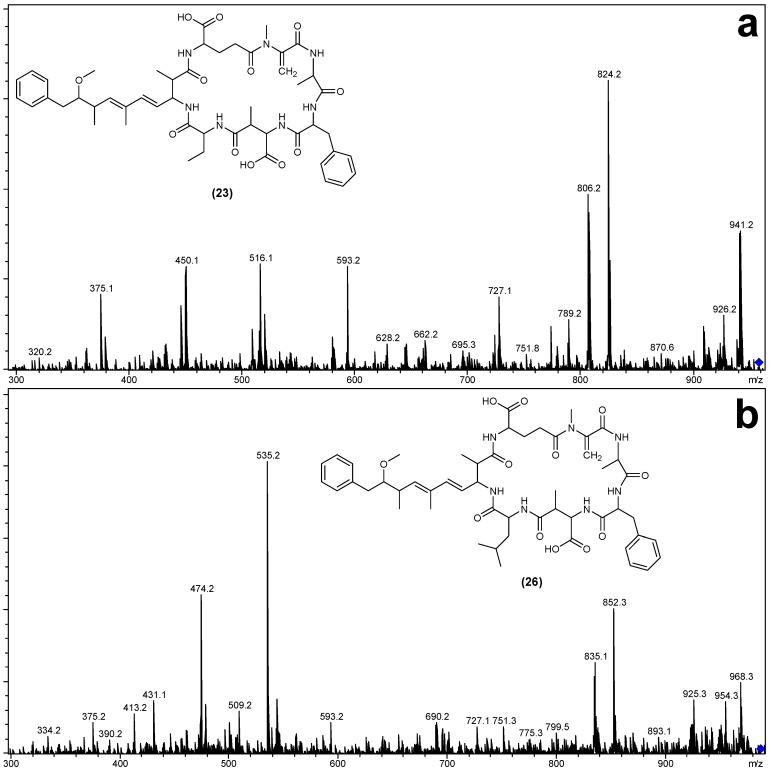
Electrospray ionization collision-induced dissociation tandem mass spectra and putative structures of MC-FAba (**a**) and MC-FL (**b**).

The MS/MS spectra of **23** and **26** (Figure 4) indicated that they were very similar in structure to MC-FA (**19**), except that the fragments attributed to the position four amino acid contained either 14 or 42 Da additional mass (Table 2). The most likely substitution to cause these mass deviations was the incorporation of Aba into **23** (MC-FAba) and leucine into **26** (MC-FL), in place of the alanine observed in **19** (MC-FA).

**Figure 5:**
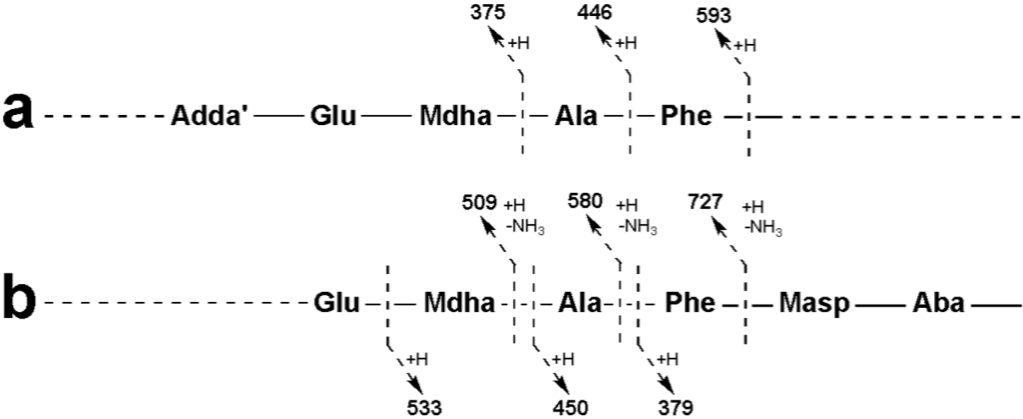
Tandem mass spectrometry fragment ions indicating the amino acid sequence in MC-FAba.

For MC-FAba (**23**), the fragment ion series starting with Adda′-Glu-Mdha (*m/z* 375) was extended to include Ala and Phe (Figure 5a). This sequence was supported by the ion series containing Adda minus NH_3_ (*m/z* 509, 580 and 727; Figure 5b). A fragment ion series which began with Phe-Masp-Aba (*m/z* 379; Figure 5b) and extended in the opposite direction to include Ala and Mdha gave the complete amino acid sequence of Adda-Glu-Mdha-Ala-Phe-Masp-Aba. A fragment resulting from the loss of Mdha and water (*m/z* 857; Table 2) indicated that Adda and the Aba residue were joined and that the structure was cyclic. The amino acid sequence in MC-FL (**26**) was similarly established.

**Table 2:** Electrospray ionization collision-induced dissociation tandem mass spectrometry fragment ions observed for MC-FA, MC-FAba and MC-FL.

Fragment Assignment *^a^*	MC-FA (19)	MC-FAba (23)	MC-FL (26)
M + H	944	958	986
M − H_2_O + H	926	940	968
M − Mdha − H_2_O + H	843	857	885
M − Adda sidechain + H	810	824	852
M − Adda sidechain − H_2_O + H	792	806	834
M − Adda + H	631	645	673
M − Adda − H_2_O + H	613	627	655
Adda-Glu-Mdha-Ala-Phe-Masp − NH_3_ + H	856	856	856
Adda-Glu-Mdha-Ala-Phe − NH_3_ + H	727	727	727
Adda-Glu-Mdha-Ala − NH_3_ + H	580	580	580
Adda-Glu-Mdha − NH_3_ + H	509	509	509
Adda′-Glu-Mdha-Ala-Phe + H	593	593	593
Adda′-Glu-Mdha-Ala + H	446	446	446
Adda′-Glu-Mdha + H	375	375	375
Mdha-Ala-Phe-Masp-*Z* + NH_4_	471	533	561
Ala-Phe-Masp-*Z* + NH_4_	388	450	478
Phe-Masp-*Z* + NH_4_	317	379	407
Mdha-Ala-Phe-Masp-*Z* + H	454	516	544
Ala-Phe-Masp-*Z* + H	371	433	461
Phe-Masp-*Z* + H	300	362	390

*^a^* *Z* = Position four amino acid (for **19** = 71 Da, **23** = 85 Da and **26** = 113 Da); Adda′ = Adda minus NH_2_ and the sidechain (C_9_H_11_O).

**Figure 6:**
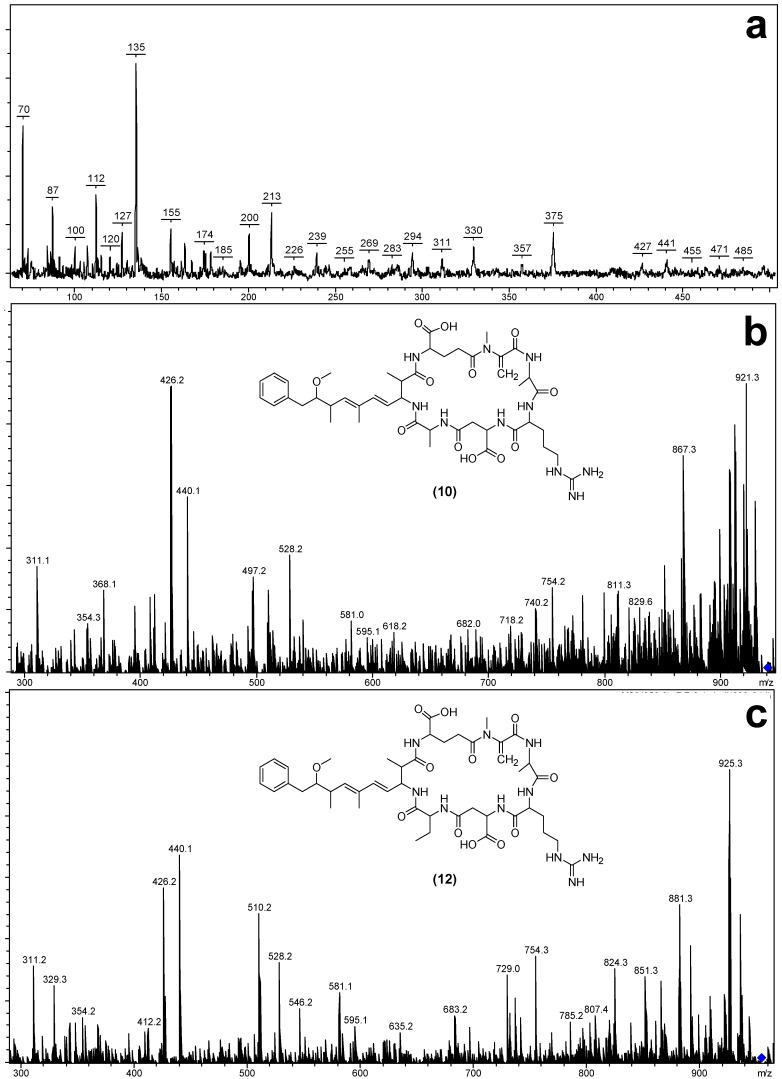
Matrix-assisted laser desorption/ionization post-source decay mass spectrum of [Asp^3^] MC-RA (**a**) and electrospray ionization collision-induced dissociation tandem mass spectra with putative structures of [Asp^3^] MC-RA (**b**) and [Asp^3^] MC-RAba (**c**).

The MS/MS spectra of **10** and **12** (Figure 6) indicated that they had structures similar to the known microcystins, MC-RA (**11**) and MC-RAba (**13**) respectively, except that the fragments attributed to the position three Masp contained 14 Da less mass (Table 3). The most likely substitution to cause this mass deviation was the presence of aspartic acid in **10** ([Asp^3^] MC-RA) and **12** ([Asp^3^] MC-RAba) in place of the Masp observed in **11** and **13**.

**Table 3:** Matrix-assisted laser desorption/ionization post-source decay (MALDI PSD) and electrospray ionization collision-induced dissociation (ESI CID) tandem mass spectrometry fragment ions observed for [Asp^3^] MC-RA, MC-RA, [Asp^3^] MC-RAba and MC-RAba.

Fragment Assignment *^a^*	[Asp^3^] MC-RA (10)	MC-RA (11)	[Asp^3^] MC-RAba (12)	MC-Raba (13)
M + H *^b,c^*	939	953	953	967
M − H_2_O + H *^b,c^*	921	935	935	949
M − COOH + H *^c^*	894	908	908	922
M − *Z* + H *^c^*	868	882	868	882
M − CH_2_NHCN_2_H_3_ + H *^c^*	867	881	881	895
M − Glu + H *^c^*	810	824	824	838
M − (Me)Asp + H *^c^*	824	824	838	838
M − Adda sidechain + H *^c^*	787	819	819	833
Mdha-Ala-Arg-(Me)Asp-*Z* + NH_4_ *^c^*	514	528	528	542
Mdha-Ala-Arg-(Me)Asp-*Z* − H_2_O + NH_4_ *^c^*	496	510	510	524
Mdha-Ala-Arg-(Me)Asp-*Z* + H *^c^*	497	511	511	525
Mdha-Ala-Arg-(Me)Asp − CH_2_NHCN_2_H_3_ + H *^c^*	354	368	354	368
Mdha-Ala-Arg-(Me)Asp + H *^c^*	426	440	426	440
Mdha-Ala-Arg + H *^b,c^*	311	311	311	311
Mdha-Ala + H *^b^*	155	155	-	155
Arg-(Me)Asp-*Z* + H *^c^*	343	357	357	
Glu-Mdha-Ala-Arg − COOH + H *^c^*	395	395	395	395
Glu-Mdha-Ala-Arg − CH_2_NHCN_2_H_3_ + H *^c^*	368	368	368	368
Glu-Mdha-Ala-Arg + H *^c^*	440	440	440	440
Glu-Mdha + H *^b^*		213	-	213
Adda′-Glu-Mdha + H *^b,c^*	375	375	375	375
Adda′ + H *^b^*	163	163	-	163

*^a^* *Z* = Position four amino acid (for **10**–**11** = 71 Da and **12**–**13** = 85 Da); Adda′ = Adda minus NH_2_ and the sidechain (C_9_H_11_O); CH_2_NHCN_2_H_3_ is a fragment of the arginine sidechain; *^b^* Fragments observed in the MALDI PSD spectra; *^c^* Fragments observed in the ESI CID spectra. Note: MALDI PSD analysis could not be conducted on **12**.

**Figure 7:**
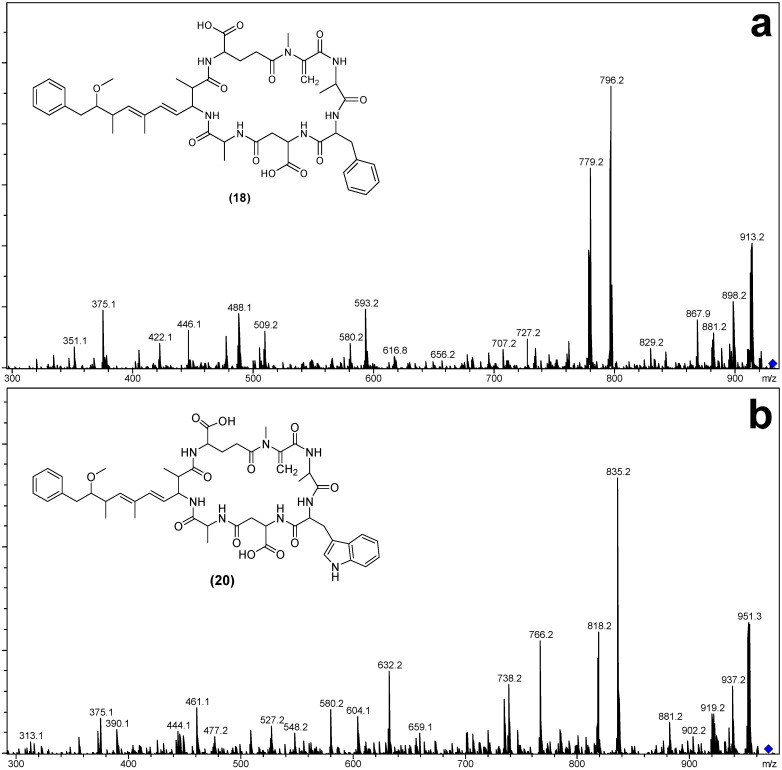
Electrospray ionization collision-induced dissociation tandem mass spectra and putative structures of [Asp^3^] MC-FA (**a**) and [Asp^3^] MC-WA (**b**).

Similarly, the MS/MS spectra of **18** and **20** (Figure 7) indicated that they had structures similar to the known microcystins, MC-FA (**19**) and MC-WA (**21**) respectively, except that the fragments attributed to the position three Masp contained 14 Da less mass (Table 4). Again, the most likely substitution to cause this mass deviation was the presence of aspartic acid in **18** ([Asp^3^] MC-FA) and **20** ([Asp^3^] MC-WA) in place of the Masp observed in **19** and **21**.

The identification and putative characterization of six new microcystins (**10**, **12**, **18**, **20**, **23** and **26**) from *Microcystis* CAWBG11 is a notable increase to the ≥100 different microcystin congeners characterized to date [6]. As all of the new congeners reported here were present at low levels, characterization was conducted by mass spectrometric methods and chemical derivatization. Two of the new microcystins (**23** and **26**) followed the general structure of a microcystin, consisting of Adda, Glu, Mdha, Ala and Masp, with variable amino acids in positions two and four. These congeners contained phenylalanine in position two with 2-aminobutanoic acid (**23**) or leucine (**26**) in position four. The remaining four new microcystins (**10**, **12**, **18** and **20**) were [Asp^3^] desmethyl analogues of previously identified congeners.

Four of the new congeners were hydrophobic microcystins (**18**, **20**, **23** and **26**) containing neutral amino acids in positions two and four. Two of the new congeners (**10** and **12**) contained position two arginine residues in conjunction with a neutral amino acid in position four (MC-RZs). Previously, the only -RZ microcystin reported was MC-RA [38], although recently more of these congeners have been identified [39,49,52,55].

**Table 4:** Electrospray ionization collision-induced dissociation tandem mass spectrometry fragment ions observed for [Asp^3^] MC-FA, MC-FA, [Asp^3^] MC-WA and MC-WA.

Fragment Assignment *^a^*	[Asp^3^] MC-FA (18)	MC-FA (19)	[Asp^3^] MC-WA (20)	MC-WA (21)
M + H	930	944	969	983
M − H_2_O + H	912	926	951	965
M − Mdha − H_2_O + H	829	843	868	882
M − Adda sidechain + H	796	810	835	849
M − Adda sidechain − H_2_O + H	778	792	817	831
M − Adda + H	617	631	656	670
M − Adda − H_2_O + H	599	613	638	652
Adda-Glu-Mdha-Ala-*X*-(Me)Asp − NH_3_ + H	842	856	881	895
Adda-Glu-Mdha-Ala-*X* − NH_3_ + H	727	727	766	766
Adda-Glu-Mdha-Ala − NH_3_ + H	580	580	580	580
Adda-Glu-Mdha − NH_3_ + H	509	509	509	509
Adda′-Glu-Mdha-Ala-*X* + H	593	593	632	632
Adda′-Glu-Mdha-Ala + H	446	446	446	446
Adda′-Glu-Mdha + H	375	375	375	375
Mdha-Ala-*X*-(Me)Asp-Ala + NH_4_	505	519		558
Ala-*X*-(Me)Asp-Ala + NH_4_	422	436	461	475
*X*-(Me)Asp-Ala + NH_4_	351	365	390	404
Mdha-Ala-*X*-(Me)Asp-Ala + H	334	502	527	541
Ala-*X*-(Me)Asp-Ala + H	405	419	444	458
*X*-(Me)Asp-Ala + H	488	348	373	387

*^a^* *X* = Position two amino acid (for **18**–**19** = 147 Da and **20**–**21** = 186 Da); Adda′ = Adda minus NH_2_ and the sidechain (C_9_H_11_O).

An interesting feature of the -RZ microcystin congeners is that whilst the amino acid composition may be identical to an -XR microcystin, the -RZ congeners are retained for longer during reversed-phase C_18_ chromatography (see Table 1). This observation has been noted by others [38,52] and provides a straightforward means of differentiating between -XR and -RZ microcystin congeners. The change in retention time of the -RZ microcystins is most likely due to the position five Adda moiety being the major hydrophobic region of a microcystin. As a result, the alkyl chains of the C_18_ column packing are likely to interact predominantly with this region of the compound. When an Arg residue is present in position four next to the Adda moiety (-XR microcystins), it would also interact substantially with the alkyl chains of the packing material. When an Arg residue is present in position two (-RZ microcystins) and is no longer located next to the Adda moiety, the hydrophilic effect of the protonated guanidinium group would not be as pronounced and the microcystin would be more retained on the column.

As the quantities of new microcystins were very low, no toxicology or protein phosphatase inhibition studies were conducted. However, the Adda-Glu portion of the microcystin structure, which has previously been shown to be important for protein phosphatase inhibition [7] is not modified in these congeners. Therefore, it is likely that these microcystins would inhibit protein phosphatases and pose a health risk to humans and animals.

### 2.4. The Microcystin Diversity of Microcystis CAWBG11

In this study, 27 different microcystin congeners were detected in cultures of *Microcystis* CAWBG11, due to relaxed substrate specificity of the microcystin synthase at positions two, three and four (Table 1). This indicates that the microcystin synthase in *Microcystis* CAWBG11 has relaxed substrate specificity at McyB1 as well as at McyC (see [8] for more details). The presence of position three desmethyl congeners indicates that the adenylation domain of McyB2 recognizes both Masp and Asp and results in approximately 2.5% of each microcystin congener containing Asp in position three instead of the usual Masp. Five different amino acids were observed to be incorporated into position two of the structure, two amino acids were observed in position three and four amino acids were observed in position four. This equates to a potential 40 microcystins which could be produced by CAWBG11. This value would increase if the CAWBG11 microcystin synthase were shown to be able to incorporate more amino acids than reported in the present study. As the various amino acid substitutions occur at different frequencies, not all of the possible congeners were observed in the present study, namely, [Asp^3^] analogues of MC-RL, and the -XAba, -XL and -YZ microcystin congeners. Tyrosine-containing analogues of the -XAba and -XL congeners were also not observed.

In order to better understand whether the number of microcystin congeners observed in *Microcystis* CAWBG11 was unique, relevant literature was surveyed to assess the diversity of congeners produced by isolated cyanobacterial strains (Supplementary Information Table S1). As this survey was formulated from multiple studies by different researchers, it is difficult to ascertain the level of characterization performed on each reported strain which could bias the results towards a lower microcystin diversity. In several cases, researchers were not able to identify all of the microcystins present and listed these as “unidentified microcystins” [14,43,49,56]. These “unidentified microcystins” skew the analysis slightly as it cannot be predicted whether these congeners were similar analogues to those identified or whether they were structurally different.

The investigation revealed that the median number of microcystin congeners produced by a cyanobacterial strain was between four and five (Supplementary Information Figure S3). When assessing the number of congeners identified (within the study), the median was slightly lower (Median = 4) than assessments based on the number of congeners observed (including “unidentified microcystins”; Median = 5). Besides *Microcystis* CAWBG11, the range for the number of microcystin congeners identified in a single cyanobacterial strain spanned from 1–16 (Supplementary Information Table S1), but could be extended to 47 when the unidentified congeners were taken into account.

When there were no “unidentified microcystins” present in a cyanobacterial strain, the potential number of congeners which could be produced was calculated. This took account of all of the combinations of the amino acids incorporated into positions two and four, as well as the variable modifications observed. Values for the potential microcystin production of an isolated cyanobacterial strain ranged from 1–72, with a median of four congeners. As expected, the range for the potential number of microcystins was higher than that based on the microcystins identified/observed, since some congeners occur at lower levels and may not be detected.

The number of microcystins identified in *Microcystis* CAWBG11 (27 congeners) and the potential number of microcystins which could be produced (40 congeners), positioned CAWBG11 in the top ten percentile in the distribution of strains assessed. Other strains in the top ten percentile included *Anabaena* 66A [14], *Microcystis viridis* NIES102 [14], *Nostoc* 152 [14,57,58,59] and *Nostoc* IO-102-1 [14,60]. These cyanobacterial strains were able to incorporate an array of amino acids into position two and produced variable modifications at other positions in the structure (Supplementary Information Table S1). However, these strains did not show much variability at the position four amino acid, as *Anabaena* 66A, *Microcystis viridis* NIES102 and *Nostoc* IO-102-1 could only incorporate arginine whilst *Nostoc* 152 was able to utilize arginine and homoarginine. The microcystins produced by *Microcystis* CAWBG11 contained arginine, alanine, aminobutanoic acid and leucine in position four.

Overall, amongst the 49 cyanobacterial strains assessed in the survey, there was a low degree of variability in the amino acids incorporated into position four of the microcystin structure. The majority of the strains assessed were only able to incorporate one or two amino acids into this position (Supplementary Information Table S1). Interestingly, when strains did exhibit high levels of amino acid variability at position four, they tended to exhibit a high degree of substrate specificity at position two. *Microcystis* CAWBG11, however, showed relaxed substrate specificity at both positions two and four. As a result, CAWBG11 produced microcystin congeners which contained no arginine residues, a single arginine residue and two arginine residues. Most of the other cyanobacterial strains assessed produced congeners containing one or two arginine residues or congeners that contained one or no arginine residues. Recently, three cyanobacterial strains which produced the same nine microcystins were reported: *Planktothrix agardhii* CYA 56/3, CYA 137 and CYA 532 [49]. Like CAWBG11, these strains were able to simultaneously produce microcystins which contained one, two and no arginine residues, although they exhibited a lesser degree of structural variability than CAWBG11. As CAWBG11 produces such a diverse range of microcystin congeners, it could be a useful cyanobacterial strain for investigation of the parameters which cause modulation of microcystin congener concentrations.

## 3. Experimental Section

### 3.1. Microcystis CAWBG11

*Microcystis* species CAWBG11 was isolated from a bloom sample obtained from Lake Hakanoa (Huntly, New Zealand) in 2005. A culture was initiated from a small colony (<10 cells) [22]. The culture is maintained alive and cryopreserved in the Cawthron Institute micro-algae culture collection. Based on morphological features, the strain most closely matches the descriptions of *Microcystis* *aeruginosa.* The cells are sub-spherical in shape and very densely aggregated in large colonies which are irregular in shape and have colorless, homogeneous mucilage that does not extend beyond colony edges (Supplementary Information Figure S4). Cells are bright green, with aerotopes. The 16S ribosomal RNA gene partial sequence and full 16S-23S rRNA intergenic spacer sequence are available on GenBank (EF634465). CAWBG11 has a high (>99%) sequence homology to other *Microcystis* *aeruginosa* strains (e.g., AM778951 or HF678510). Although not initiated from a single cell, the toxin profile has remained stable despite nearly 10 years of sub-culturing, thus we believe the original colony comprised a single strain.

### 3.2. General Experimental Procedures

The HRESIMS analysis was performed on a Bruker MicrOTOF mass spectrometer. Data for MALDI-TOF MS and PSD was collected on a Bruker AutoFlex II mass spectrometer. The LC-MS and LC-MS/MS analyses were performed on a Bruker AmaZon X ESI mass spectrometer coupled to a Dionex UltiMate 3000 HPLC system. Ultraviolet (UV) absorption data for purified microcystins was determined using a Cary 100 Scan UV/visible spectrophotometer (Varian) over a wavelength range of 200–800 nm. Optical rotations of purified microcystins were determined using an AUTOPOL IV polarimeter (Rudolph Research Analytical). Reversed-phased C_18_ separations were conducted using YMC-gel ODS-A (YMC) and size exclusion chromatography was conducted using Sephadex LH-20 (Pharmacia Fine Chemicals). HPLC purification was performed using Waters 515 HPLC pumps coupled to a photodiode array detector (200–400 nm; Waters 2996) and an Econosil C_18_ Column (250 × 10 mm, 10-μm; Alltech).

### 3.3. Matrix-Assisted Laser Desorption/Ionization-Time of Flight Mass Spectrometry Analysis

Samples were prepared for MALDI-TOF MS analysis by mixing an aliquot (0.5 µL) 1:1 with a saturated solution of α-cyano-4-hydroxycinnamic acid in 2:1:1 methanol/acetonitrile/0.1% trifluoroacetic acid, directly on a 600-μm anchorchip. The dried spot was washed by pipetting wash solution (5 µL; 0.1% trifluoroacetic acid in 10 mM NH_4_H_2_PO_4_) onto the spot for 5 s, then withdrawing the liquid. Spectra were acquired over various *m/z* ranges, with an acceleration voltage of 19 kV and a reflector voltage of 20 kV. Pulsed ion extraction of 60 ns was used to build up the concentration of ions in the ion source and ions below 500 *m/z* were suppressed to avoid detector saturation from matrix ions. Mass calibration was performed using a peptide calibration standard (Bruker Daltonics) which was prepared in the same manner as the samples. Post-source decay spectra were obtained from the singly-protonated ions of the target compounds.

### 3.4. Liquid Chromatography-Mass Spectrometry Analysis

Samples (20 µL) for LC-MS and LC-MS/MS were separated on a C_18_ column (Ascentis Express C_18_, 100 × 2.1 mm, 2.7-μm; Supleco Analytical) at a flow of 200 µL/min using a gradient of 2% acetonitrile + 0.1% formic acid (v/v; solvent A) and 98% acetonitrile + 0.1% formic acid (v/v; solvent B) with the following gradient program; the sample was loaded in 10% B; 10% B was held for 1 min and increased to 100% B over 12 min; 100% B was held for 2 min; the solvent composition was returned to 10% B in 1 min and the column re-equilibrated for 4 min. The eluting compounds were ionized using a capillary voltage of 3.5 kV and a nebulizer pressure of 3.0 bar. Desolvation was accomplished with a nitrogen flow of 8 L/min at 220 °C. Mass spectra were acquired for positive or negative ions over a range of *m/z* 100–2000. Daughter ion scans were obtained from the singly-protonated ions of the target compounds by CID (collision amplitude of 1.0).

### 3.5. β-mercaptoethanol Derivatization for Mdha/Dhb Determination

A recently developed thiol derivatization technique [49] was used to determine which of the isometric amino acids, Mdha or Dhb, was present in CAWBG11 microcystins. A methanol extract of CAWBG11 (1.42 mL) was mixed with 200 mM sodium bicarbonate (pH 9.7; 360 µL) in a septum-capped vial and left to equilibrate at 30 °C. Following LC-MS analysis of the original extract, β-mercaptoethanol (20 µL) was added to the extract and the vial inverted to mix. The reaction mixture was maintained at 30 °C in the sample tray of the LC-MS and injections were made periodically over a 6 h period.

### 3.6. Isolation of MC-RA and MC-RAba

*Microcystis* sp. CAWBG11 was grown in 20 × 20 L plastic carboys, each containing 16 L of MLA media [61]. Cultures were grown at 18 °C under a 12:12 h light/dark cycle with a photon-flux of 100 μE·m^−2^·s^−1^. After 40 days, the cultures were harvested using plankton netting (11-µm mesh). The concentrated cell material was lyophilized and stored at −20 °C until extracted.

Freeze-dried cells (76.9 g) were extracted in 7:3 ethanol/water (5 × 800 mL). The remaining cell pellet was extracted in methanol (5 × 250 mL). A voucher of the cellular material extracted (JP2-033-05) is held at the Department of Chemistry, University of Waikato, Hamilton, New Zealand. The crude extracts (5.6 g and 0.45 g respectively) were evaporated and individually fractionated by reversed-phase C_18_ chromatography (50 g) using a steep stepped gradient from water to methanol to dichloromethane, where **10**–**14** eluted between 3:7 and 1:1 methanol/water.

These fractions were combined (130 mg) and separated on a reversed-phase C_18_ column (20 g) acidified with 0.1% formic acid (v/v) using a steep stepped gradient from acidified water to acidified methanol to methanol to dichloromethane, where **10**–**14** eluted with 13:7 methanol/water + 0.1% formic acid (v/v).

The fraction containing **10**–**14** (36.2 mg) was dissolved in methanol and subjected to size exclusion chromatography to remove residual pigments, before the -RZ microcystins (12.5 mg) were separated by isocratic HPLC using acetonitrile:10 mM ammonium acetate (13:37). The dried samples were lyophilized then residual ammonium acetate was removed by passing the sample (dissolved in 10% methanol; v/v), through a plug of C_18_ material (200 mg) and eluting with 70% methanol (v/v) to yield **10** (<0.1 mg), **11** (0.2 mg), **12** (<0.1 mg), **13** (0.1 mg) and **14** (<0.1 mg).

**MC-RA** (**11**): White amorphous solid (0.2 mg, 2.60 × 10^−4^%); [α]^20^_D_ −100° (*c* 0.02 g/100 mL, methanol); UV (methanol) λ_max_ (log ɛ) 205 (4.26), 238 (4.26) nm; HRESIMS *m/z* 953.5122 (calculated for C_46_H_69_N_10_O_12_, 953.5091, Δ + 3.28 ppm).

**MC-RAba** (**13**): White amorphous solid (0.1 mg, 1.30 × 10^−4^%); [α]^20^_D_ −60° (*c* 0.013 g/100 mL, methanol); UV (methanol) λ_max_ (log ɛ) 207 (4.29), 238 (4.18) nm; HRESIMS *m/z* 967.5259 (calculated for C_47_H_71_N_10_O_12_, 967.5247, Δ + 1.15 ppm).

### 3.7. Advanced Marfey’s Amino Acid Analysis

Purified microcystins (**11** and **13**) were subjected to amino acid analysis using the Advanced Marfey’s method [53,62]. 1-Fluoro-2,4-dinitrophenyl-5-leucine (FDLA) was synthesized according to the method of Marfey [63], but using leucinamide (Bachem) instead of alaninamide. Both the d- and l- forms of the reagent were synthesized from the respective stereoisomers of leucinamide. Microcystins (100 µg) were dried at 35 °C under a stream of nitrogen gas, resuspended in 6 N hydrochloric acid (0.5 mL) and incubated at 110 °C for 16 h. The hydrochloric acid was removed at 35 °C under a stream of nitrogen gas. Hydrolyzates were resuspended in water (105 µL) and aliquots were placed in two microcentrifuge tubes (50 µL each), to which 1 M sodium bicarbonate (20 µL) and 1% l- or dl-FDLA (w/v; 100 µL) was added. The tubes were incubated at 40 °C for 1 h, before being quenched with 1 N hydrochloric acid (20 µL). The derivatized hydrolyzates were diluted with MeOH (810 µL), centrifuged (14,000× *g*, 5 min) and the supernatant transferred to a septum capped LC vial. The derivatized sample (20 µL) was analyzed by LC-MS using an Econosil C_18_ column (250 × 3.2 mm, 5-µm; Alltech) and a gradient of 25%–75% (v/v) acetronitrile + 0.1% (v/v) formic acid over 30 min. Eluting derivatives were detected by UV absorption (250–500 nm) and ESI MS (negative ion mode, *m/z* 300–1100).

Retention times of the l-FDLA derivatives were as follows: **11**: d-Glu (14.3 min), l-Ala (15.8 min), d-Masp (15.9 min), d-Ala (18.6 min), *N*-methylamine (19.6 min), l-Arg (27.2 min), 3(*S*)-Adda (32.9 min); **13**: d-Glu (14.3 min), d-Masp (15.9 min), l-Aba (17.4 min), d-Ala (18.6 min), *N*-methylamine (19.6 min), l-Arg (27.2 min), 3(*S*)-Adda (32.9 min).

## 4. Conclusions

Assessment of *Microcystis* CAWBG11 indicated the presence of numerous oligopeptides including aeruginosins, microviridins and microcystins. Further investigation of the microcystin diversity indicated that CAWBG11 produced at least 27 microcystin congeners, of which six have not been reported previously. The putative structures of [Asp^3^] MC-RA (**10**), [Asp^3^] MC-RAba (**12**), MC-FAba (**18**), MC-FL (**20**), [Asp^3^] MC-FA (**23**) and [Asp^3^] MC-WA (**26**) were determined by MS/MS and thiol derivatization. The number of microcystin congeners produced by CAWBG11 was in the upper 10^th^ percentile of the cyanobacterial strains assessed. Uniquely, CAWBG11 showed combined fluidity of the amino acids incorporated into positions two and four which allows it to simultaneously produce congeners containing no arginine residues, a single arginine residue and two arginine residues. This has only recently been reported in other cyanobacterial strains [49].

## Supplementary Files

Supplementary File 1

## References

[1] Holt J.G., Krieg N.R., Sneath P.H.A., Staley J.T., Williams S.T. (2000). Oxygenic photosynthetic bacteria. Bergey’s Manual of Determinative Bacteriology.

[2] Whitton B.A., Potts M. (2000). The Ecology of Cyanobacteria, Their Diversity in Time and Space.

[3] Jones G.J., Korth W. (1995). *In situ* production of volatile odour compounds by river and reservoir phytoplankton populations in Australia. Water Sci. Technol..

[4] Sivonen K., Jones G., Chorus I., Bartram J. (1999). Cyanobacterial toxins. Toxic Cyanobacteria in Water: A Guide to Their Public Health Consequences, Monitoring and Management.

[5] Van Apeldoorn M.E., van Egmond H.P., Speijers G.J.A., Bakker G.J.I. (2007). Toxins of cyanobacteria. Mol. Nutr. Food Res..

[6] Niedermeyer T. (2013). Microcystin Congeners Described in the Literature. http://dx.doi.org/10.6084/m9.figshare.880756.

[7] Rinehart K., Namikoshi M., Choi B. (1994). Structure and biosynthesis of toxins from blue-green algae (cyanobacteria). J. Appl. Phycol..

[8] Tillett D., Dittmann E., Erhard M., von Döhren H., Börner T., Neilan B.A. (2000). Structural organization of microcystin biosynthesis in *Microcystis aeruginosa* PCC7806: An integrated peptide-polyketide synthetase system. Chem. Biol..

[9] Moore R.E., Chen J.L., Moore B.S., Patterson G.M.L., Carmichael W.W. (1991). Biosynthesis of microcystin-LR: Origin of the carbons in the Adda and Masp units. J. Am. Chem. Soc..

[10] Nishizawa T., Asayama M., Fujii K., Harada K.-I., Shirai M. (1999). Genetic analysis of the peptide synthetase genes for a cyclic heptapeptide microcystin in *Microcystis* spp.. J. Biochem..

[11] Nishizawa T., Ueda A., Asayama M., Fujii K., Harada K.-I., Ochi K., Shirai M. (2000). Polyketide synthase gene coupled to the peptide synthetase module involved in the biosynthesis of the cyclic heptapeptide microcystin. J. Biochem..

[12] Börner T., Dittmann E., Huisman J., Matthijs H.C.P., Visser P.M. (2005). Molecular biology of cyanobacterial toxins. Harmful Cyanobacteria.

[13] Mikalsen B., Boison G., Skulberg O.M., Fastner J., Davies W., Gabrielsen T.M., Rudi K., Jakobsen K.S. (2003). Natural variation in the microcystin synthetase operon *mcyABC* and impact on microcystin production in *Microcystis* strains. J. Bacteriol..

[14] Fewer D., Rouhiainen L., Jokela J., Wahlsten M., Laakso K., Wang H., Sivonen K. (2007). Recurrent adenylation domain replacement in the microcystin synthetase gene cluster. BMC Evol. Biol..

[15] Fischer A., Hoeger S.J., Stemmer K., Feurstein D.J., Knobeloch D., Nussler A., Dietrich D.R. (2010). The role of organic anion transporting polypeptides (OATPs/SLCOs) in the toxicity of different microcystin congeners *in vitro*: A comparison of primary human hepatocytes and OATP-transfected HEK293 cells. Toxicol. Appl. Pharmacol..

[16] An J., Carmichael W.W. (1994). Use of a colorimetric protein phosphatase inhibition assay and enzyme linked immunosorbent assay for the study of microcystins and nodularins. Toxicon.

[17] Runnegar M.T., Falconer I.R., Silver J. (1981). Deformation of isolated rat hepatocytes by a peptide hepatotoxin from the blue-green alga *Microcystis* aeruginosa. Naunyn-Schmiedeberg’s Arch. Pharmacol..

[18] Falconer I.R., Yeung D.S.K. (1992). Cytoskeletal changes in hepatocytes induced by *Microcystis* toxins and their relation to hyperphosphorylation of cell proteins. Chem. Biol. Interact..

[19] Fujiki H., Suganuma M. (1993). Tumor promotion by inhibitors of protein phosphatases 1 and 2A: The okadaic acid class of compounds. Adv. Cancer Res..

[20] Sainis I., Fokas D., Vareli K., Tzakos A., Kounnis V., Briasoulis E. (2010). Cyanobacterial cyclopeptides as lead compounds to novel targeted cancer drugs. Mar. Drugs.

[21] Niedermeyer T.H.J., Daily A., Swiatecka-Hagenbruch M., Moscow J.A. (2014). Selectivity and potency of microcystin congeners against OATP1B1 and OATP1B3 expressing cancer cells. PLoS One.

[22] Rueckert A., Wood S.A., Cary S.C. (2007). Development and field assessment of a quantitative PCR for the detection and enumeration of the noxious bloom-former Anabaena planktonica. Limnol. Oceanogr. Methods.

[23] Ishida K., Okita Y., Matsuda H., Okino T., Murakami M. (1999). Aeruginosins, protease inhibitors from the cyanobacterium *Microcystis* aeruginosa. Tetrahedron.

[24] Ploutno A., Carmeli S. (2002). Modified peptides from a water bloom of the cyanobacterium *Nostoc* sp.. Tetrahedron.

[25] Welker M., Marsálek B., Sejnohová L., von Döhren H. (2006). Detection and identification of oligopeptides in *Microcystis* (cyanobacteria) colonies: Toward an understanding of metabolic diversity. Peptides.

[26] Murakami M., Okita Y., Matsuda H., Okino T., Yamaguchi K. (1994). Aeruginosin 298-A, a thrombin and trypsin inhibitor from the blue-green alga Microcystis aeruginosa (NIES-298). Tetrahedron Lett..

[27] Howard K.L., Boyer G.L. (2007). Quantitative analysis of cyanobacterial toxins by matrix-assisted laser desorption ionization mass spectrometry. Anal. Chem..

[28] Puddick J., Prinsep M.R., Wood S.A., Miles C.O., Rise F., Cary S.C., Hamilton D.P., Wilkins A.L. (2013). Structural characterization of new microcystins containing tryptophan and oxidized tryptophan residues. Mar. Drugs.

[29] Meriluoto J.A.O., Sandström A., Eriksson J.E., Remaud G., Grey Graig A., Chattopadhyaya J. (1989). Structure and toxicity of a peptide hepatotoxin from the cyanobacterium *Oscillatoria agardhii*. Toxicon.

[30] Kusumi T., Ooi T., Watanabe M.M., Takahashi H., Kakisawa H. (1987). Cyanoviridin RR, a toxin from the cyanobacterium (blue-green alga) Microcystis viridis. Tetrahedron Lett..

[31] Painuly P., Perez R., Fukai T., Shimizu Y. (1988). The structure of a cyclic peptide toxin, cyanogenosin-RR from Microcystis aeruginosa. Tetrahedron Lett..

[32] Watanabe M.F., Oishi S., Harada K.-I., Matsuura K., Kawai H., Suzuki M. (1988). Toxins contained in *Microcystis* species of cyanobacteria (blue-green algae). Toxicon.

[33] Botes D.P., Wessels P.L., Kruger H., Runnegar M.T.C., Santikarn S., Smith R.J., Barna J.C.J., Williams D.H. (1985). Structural studies on cyanoginosins-LR, -YR, -YA, and -YM, peptide toxins from Microcystis aeruginosa. Org. Bioorg. Chem..

[34] Krishnamurthy T., Szafraniec L., Hunt D.F., Shabanowitz J., Yates J.R., Hauer C.R., Carmichael W.W., Skulberg O., Codd G.A., Missler S. (1989). Structural characterization of toxic cyclic peptides from blue-green algae by tandem mass spectrometry. Proc. Natl. Acad. Sci. USA.

[35] Harada K.-I., Ogawa K., Kimura Y., Murata H., Suzuki M., Thorn P.M., Evans W.R., Carmichael W.W. (1991). Microcystins from *Anabaena flos-aquae* NRC 525-17. Chem. Res. Toxicol..

[36] Lee T.-H., Chou H.-N. (2000). Isolation and identification of seven microcystins from a cultured M.TN-2 strain of Microcystis aeruginosa. Bot. Bull. Academ. Sinica.

[37] Namikoshi M., Rinehart K.L., Sakai R., Stotts R.R., Dahlem A.M., Beasley V.R., Carmichael W.W., Evans W.R. (1992). Identification of 12 hepatotoxins from a Homer Lake bloom of the cyanobacteria *Microcystis aeruginosa*, *Microcystis viridis*, and *Microcystis wesenbergii*: Nine new microcystins. J. Org. Chem..

[38] Lee T.-H., Chen Y.-M., Chou H.-N. (1998). First report of microcystins in Taiwan. Toxicon.

[39] Miles C.O., Sandvik M., Nonga H.E., Rundberget T., Wilkins A.L., Rise F., Ballot A. (2012). Thiol derivatization for LC-MS identification of microcystins in complex matrices. Environ. Toxicol..

[40] Diehnelt C.W., Dugan N.R., Peterman S.M., Budde W.L. (2006). Identification of microcystin toxins from a strain of *Microcystis aeruginosa* by liquid chromatography introduction into a hybrid linear ion trap-fourier transform ion cyclotron resonance mass spectrometer. Anal. Chem..

[41] Botes D.P., Tuinman A.A., Wessels P.L., Viljoen C.C., Kruger H., Williams D.H., Santikarn S., Smith R.J., Hammond S.J. (1984). The structure of cyanoginosin-LA, a cyclic heptapeptide toxin from the cyanobacterium Microcystis aeruginosa. Org. Bioorg. Chem..

[42] Puddick J., Prinsep M.R., Wood S.A., Cary S.C., Hamilton D.P., Wilkins A.L. (2013). Isolation and structure determination of two new hydrophobic microcystins from *Microcystis* sp. (CAWBG11). Phytochem. Lett..

[43] Del Campo F.F., Ouahid Y. (2010). Identification of microcystins from three collection strains of *Microcystis aeruginosa*. Environ. Pollut..

[44] Gathercole P.S., Thiel P.G. (1987). Liquid chromatographic determination of the cyanoginosins, toxins produced by the cyanobacterium *Microcystis aeruginosa*. J. Chromatogr. A.

[45] Craig M., McCready T.L., Luu H.A., Smillie M.A., Dubord P., Holmes C.F.B. (1993). Identification and characterization of hydrophobic microcystins in Canadian freshwater cyanobacteria. Toxicon.

[46] Beattie K.A., Kaya K., Sano T., Codd G.A. (1998). Three dehydrobutyrine-containing microcystins from Nostoc. Phytochemistry.

[47] Sano T., Beattie K.A., Codd G.A., Kaya K. (1998). Two (*Z*)-dehydrobutyrine-containing microcystins from a hepatotoxic bloom of *Oscillatoria agardhii* from Soulseat Loch, Scotland. J. Nat. Prod..

[48] Sano T., Kaya K. (1998). Two new (*E*)-2-amino-2-butenoic acid (Dhb)-containing microcystins isolated from *Oscillatoria agardhii*. Tetrahedron.

[49] Miles C.O., Sandvik M., Haande S., Nonga H., Ballot A. (2013). First use of LC-MS analysis with thiol derivatization to differentiate [Dhb^7^]- from [Mdha^7^]-microcystins: Analysis of cyanobacterial blooms, *Planktothrix* cultures and European crayfish from Lake Steinsfjorden, Norway. Environ. Sci. Technol..

[50] Smith J.L., Boyer G.L. (2009). Standardization of microcystin extraction from fish tissues: A novel internal standard as a surrogate for polar and non-polar variants. Toxicon.

[51] Frias H.V., Mendes M.A., Cardozo K.H.M., Carvalho V.M., Tomazela D., Colepicolo P., Pinto E. (2006). Use of electrospray tandem mass spectrometry for identification of microcystins during a cyanobacterial bloom event. Biochem. Biophys. Res. Commun..

[52] Okello W., Portmann C., Erhard M., Gademann K., Kurmayer R. (2010). Occurrence of microcystin-producing cyanobacteria in Ugandan freshwater habitats. Environ. Toxicol..

[53] Fujii K., Ikai Y., Oka H., Suzuki M., Harada K.-I. (1997). A nonempirical method using LC/MS for determination of the absolute configuration of constituent amino acids in a peptide: Combination of Marfey’s method with mass spectrometry and its practical application. Anal. Chem..

[54] Mahakhant A., Sano T., Ratanachot P., Tong-a-ram T., Srivastava V.C., Watanabe M.M., Kaya K. (1998). Detection of microcystins from cyanobacterial water blooms in Thailand fresh water. Phycol. Res..

[55] Okello W., Ostermaier V., Portmann C., Gademann K., Kurmayer R. (2010). Spatial isolation favours the divergence in microcystin net production by *Microcystis* in Ugandan freshwater lakes. Water Res..

[56] Gademann K., Portmann C., Blom J.F., Zeder M., Jüttner F. (2010). Multiple toxin production in the cyanobacterium *Microcystis*: Isolation of the toxic protease inhibitor cyanopeptolin 1020. J. Nat. Prod..

[57] Namikoshi M., Rinehart K.L., Sakai R., Sivonen K., Carmichael W.W. (1990). Structures of three new cyclic heptapeptide hepatotoxins produced by the cyanobacterium (blue-green alga) *Nostoc* sp. strain 152. J. Org. Chem..

[58] Sivonen K., Namikoshi M., Evans W.R., Fardig M., Carmichael W.W., Rinehart K.L. (1992). Three new microcystins, cyclic heptapeptide hepatotoxins, from *Nostoc* sp. strain 152. Chem. Res. Toxicol..

[59] Sivonen K., Carmichael W.W., Namikoshi M., Rinehart K.L., Dahlem A.M., Niemela S.I. (1990). Isolation and characterization of hepatotoxic microcystin homologs from the filamentous freshwater cyanobacterium *Nostoc* sp. strain 152. Appl. Environ. Microbiol..

[60] Oksanen I., Jokela J., Fewer D.P., Wahlsten M., Rikkinen J., Sivonen K. (2004). Discovery of rare and highly toxic microcystins from lichen-associated cyanobacterium *Nostoc* sp. strain IO-102-I. Appl. Environ. Microbiol..

[61] Bolch C., Blackburn S. (1996). Isolation and purification of Australian isolates of the toxic cyanobacterium; *Microcystis aeruginosa*; Kütz. J. Appl. Phycol..

[62] Fujii K., Ikai Y., Mayumi T., Oka H., Suzuki M., Harada K.-I. (1997). A nonempirical method using LC/MS for determination of the absolute configuration of constituent amino acids in a peptide: Elucidation of limitations of Marfey’s method and of its separation mechanism. Anal. Chem..

[63] Marfey P. (1984). Determination of d-amino acids. II. Use of a bifunctional reagent, 1,5-difluoro-2,4-dinitrobenzene. Carlsberg Res. Commun..

