# Supplementary material for: High Levels of Structural Diversity Observed in Microcystins from Microcystis CAWBG11 and Characterization of Six New Microcystin Congeners
Source: Mar Drugs. 2014 Nov 13;12(11):5372–95. doi: 10.3390/md12115372 (PMC4245536; doi:10.3390/md12115372)
Supplement: Supplementary File 1 [file marinedrugs-12-05372-s001.pdf]

Supplementary Information

Table S1. Microcystin (MC) congeners produced by reported cyanobacterial strains.
Table S2. Tandem mass spectrometry fragment assignments for the CAWBG11 -RR microcystin (MC) congeners observed by electrospray ionization collision-induced dissociation.
Table S3. Tandem mass spectrometry fragment assignments for the CAWBG11 -XR microcystin (MC) congeners observed by matrix-assisted laser desorption/ionization post-source decay and electrospray ionization collision-induced dissociation.
Table S4. Tandem mass spectrometry fragment assignments for the CAWBG11 -RZ microcystin (MC) congeners observed by matrix-assisted laser desorption/ionization post-source decay and electrospray ionization collision-induced dissociation.
Table S5. Tandem mass spectrometry fragment assignments for the CAWBG11 -XA microcystin (MC) congeners observed by electrospray ionization collision-induced dissociation.
Table S6. Tandem mass spectrometry fragment assignments for the CAWBG11 -XAba microcystin (MC) congeners observed by electrospray ionization collision-induced dissociation.
Table S7. Tandem mass spectrometry fragment assignments for the CAWBG11 -XL microcystin (MC) congeners observed by electrospray ionization collision-induced dissociation.
Table S8. Electrospray high-resolution mass spectrometry data for microcystins (MC) present in sufficient quantities in *Microcystis* CAWBG11.
Figure S1. Advanced Marfey's amino acid analysis of MC-RA; extracted ion chromatograms of hydrolyzed MC-RA derivatized with L-FDLA.
Figure S2. Advanced Marfey's amino acid analysis of MC-RAba; extracted ion chromatograms of hydrolyzed MC-RAba derivatized with L-FDLA.
Figure S3. Box plots representing the spread in the number of microcystin congeners produced by reported cyanobacterial strains. Plots depict the number of microcystin congeners identified; 49 strains (a); the number of microcystin congeners observed; 49 strains (b) and the potential number of congeners which could be produced according to the reported data; 33 strains (c).
Figure S4. Microscopic images of *Microcystis* CAWBG11 acquired on an Olympus IX70 inverted microscope at 100 × magnification (a) and at 1000 × magnification (b).

Table S1. Microcystin (MC) congeners produced by reported cyanobacterial strains [a].

Cyanobacterial Strain	Microcystins Identified	Number of MCs [b]	Position Two [c]	Position Four [d]	Other Modifications	Potential MCs [e]	Reference(s)
Anabaena 18B6	[Dha^7] MC-RR; [Asp^3, Dha^7] MC-RR; Unidentified MCs ×2	4	Arg	Arg	Position 3 ×2<br>Position 7 ×1	-	[1]
Anabaena 60	MC-LR; [Asp^3] MC-LR; MC-RR; [Asp^3] MC-RR	4	Leu<br>Arg	Arg	Position 3 ×2	4	[2]
Anabaena 66	[Dha^7] MC-HphR; [Dha^7] MC-HtyR; [Ser^7] MC-HtyR; [Asp^3, Dha^7] MC-HtyR	4	Hph<br>Hty	Arg	Position 3 ×2<br>Position 7 ×2	8	[2,3]
Anabaena 66A	[Dha^7] MC-LR; [Ser^7] MC-LR; [Asp^3, Ser^7] MC-LR; [Asp^3, Dha^7] MC-LR; [Dha^7] MC-FR; [Asp^3, Dha^7] MC-FR; [Dha^7] MC-HphR; [Asp^3, Dha^7] MC-HphR; MC-HtyR; [Dha^7] MC-HtyR; [Ser^7] MC-HtyR; [Asp^3, Dha^7] MC-HtyR; [Asp^3, Ser^7] MC-HtyR; Unidentified MCs ×20	33	Leu<br>Phe<br>Hph<br>Hty	Arg	Position 3 ×2<br>Position 7 ×3	-	[1]
Anabaena 90	MC-LR; [Asp^3] MC-LR; [DMAdda^5] MC-LR; [Dha^7] MC-LR; [MeSer^7] MC-LR; [Asp^3, MeSer^7] MC-LR; MC-HilR; [Asp^3] MC-HilR; MC-RR; [Asp^3] MC-RR; [Dha^7] MC-RR	11	Leu<br>Hil<br>Arg	Arg	Position 3 ×2<br>Position 5 ×2<br>Position 7 ×3	36	[1,2,4,5]
Anabaena 141	MC-LR; [Asp^3] MC-LR; MC-RR; [Asp^3] MC-RR	4	Leu<br>Arg	Arg	Position 3 ×2	4	[2]
Anabaena 186	[Dha^7] MC-E(OMe)E(OMe); [Ser^7] MC-E(OMe)E(OMe); [Asp^3, Dha^7] MC-E(Ome)E(OMe); [Asp^3, Ser^7] MC-E(OMe)E(OMe); [Dha^7] MC-EE(OMe); [Ser^7] MC-EE(OMe); [Asp^3, Dha^7] MC-EE(OMe)	7	Glu<br>Glu(OMe)	Glu(OMe)	Position 3 ×2<br>Position 7 ×2	8	[6]
Anabaena 202 A1	[Dha^7] MC-LR; [Asp^3, Dha^7] MC-LR; [Ser^7] MC-LR; [Asp^3, Ser^7] MC-HilR; [Dha^7] MC-RR; [Asp^3, Dha^7] MC-RR; [Ser^7] MC-RR	7	Leu<br>Hil<br>Arg	Arg	Position 3 ×2<br>Position 7 ×2	12	[2,7]

Table S1. *Cont.*

Anabaena 202 A2	[Dha^7] MC-LR; [Asp^3, Dha^7] MC-LR; [Ser^7] MC-LR; [Dha^7] MC-RR; [Asp^3, Dha^7] MC-RR; [Ser^7] MC-RR	6	Leu<br>Arg	Arg	Position 3 × 2<br>Position 7 × 2	8	[2,5,7]
Anabaena flos-aquae CYA83/1	MC-LR; [Asp^3] MC-LR; MC-RR; [Asp^3] MC-RR; [Glu(OMe)^6] MC-LR; [Asp^3, Glu(OMe)^6] MC-LR	6	Leu<br>Arg	Arg	Position 3 × 2<br>Position 6 × 2	8	[8]
Anabaena flos-aquae NRC 525-17	MC-LR; [Asp^3] MC-LR; MC-HtyR; [Asp^3] MC-HtyR	4	Leu<br>Hty	Arg	Position 3 × 2	4	[9]
Fischerella CENA161	MC-LR	1	Leu	Arg		1	[10]
Hapalosiphon hibernicus BZ-3-1	MC-VA; [Asp^3] MC-VA; MC-LA; [Asp^3] MC-LA; [Dha^7] MC-LA; [Asp^3, DMAdda^5] MC-LA; MC-LV; MC-LL; MC-RA; [Asp^3] MC-RA	10	Val<br>Leu<br>Arg	Ala<br>Val<br>Leu	Position 3 × 2<br>Position 5 × 2<br>Position 7 × 2	72	[1]
Microcystis CAWBG11	MC-LA; [Asp^3] MC-LA; MC-FA; [Asp^3] MC-FA; MC-YA; MC-WA; [Asp^3] MC-WA; MC-RA; [Asp^3] MC-RA; MC-RAba; [Asp^3] MC-RAba; MC-RL; MC-LAba; MC-FAba; MC-WAba; MC-LL; MC-FL; MC-WL; MC-LR; [Asp^3] MC-LR; MC-FR; [Asp^3] MC-FR; MC-YR; MC-WR; [Asp^3] MC-WR; MC-RR; [Asp^3] MC-RR	27	Leu<br>Phe<br>Tyr<br>Trp<br>Arg	Ala<br>Aba<br>Leu<br>Arg	Position 3 × 2	40	This Study
Microcystis HUB 5-2-4	MC-LR; dmMC-LR; MC-RR; dmMC-RR; MC-YR	5	Leu<br>Tyr<br>Arg	Arg	DM × 2	6	[11]
Microcystis MB-K	MC-LR; dmMC-LR; MC-YR; dmMC-YR	4	Leu<br>Tyr	Arg	DM × 2	4	[12]
Microcystis MG-K	MC-RR; dmMC-RR; MC-WR	3	Arg<br>Trp	Arg	DM × 2	4	[12]

Table S1. *Cont.*

Microcystis PCC7806	MC-LR; [Asp^3] MC-LR; [Dha^7] MC-LR; [$MeSer^7$] MC-LR; [Asp^3, Dha^7] MC-LR; [Asp^3, $MeSer^7$] MC-LR; Unidentified MCs × 5	11	Leu	Arg	Position 3 × 2<br>Position 7 × 3	-	[1,13]
Microcystis PCC7813	MC-LR; [Asp^3] MC-LR	2	Leu	Arg	Position 3 × 2	2	[11]
Microcystis aeruginosa	MC-LR	1	Leu	Arg		1	[14]
Microcystis aeruginosa B2666	MC-LA; [Asp^3] MC-LA; MC-LAba; [Asp^3] MC-LAba; MC-LL; MC-LF; MC-LR; [$MeSer^7$] MC-LR	8	Leu	Ala<br>Aba<br>Leu<br>Phe<br>Arg	Position 3 × 2<br>Position 7 × 2	20	[15]
Microcystis aeruginosa CALU972	[Dha^7] MC-LR; [Asp^3, Dha^7] MC-LR; [Dha^7] MC-RR; [Asp^3, Dha^7] MC-RR; [Dha^7] MC-YR	5	Leu<br>Tyr<br>Arg	Arg	Position 3 × 2<br>Position 7 × 1	6	[16]
Microcystis aeruginosa K-139	[Dha^7] MC-LR; [Asp^3, Dha^7] MC-LR	2	Leu	Arg	Position 3 × 2<br>Position 7 × 1	2	[5]
Microcystis aeruginosa MK10.10	MC-VR; MC-LR; MC-HilR	3	Val<br>Leu<br>Hil	Arg		3	[17]
Microcystis aeruginosa NIES90	MC-LR; MC-YR; MC-RR	3	Leu<br>Tyr<br>Arg	Arg		3	[18]

Table S1. *Cont.*

Microcystis aeruginosa PCC7820	MC-LR; dmMC-LR; [Glu(OMe)^6] MC-LR; MC-LF; dmMC-LF; MC-LW; dmMC-LW; MC-LL; MC-LM; MC-LY	10	Leu	Arg<br>Phe<br>Leu<br>Met<br>Trp<br>Tyr	DM × 2<br>Position 6 × 2	24	[19]
Microcystis aeruginosa TN-2	MC-LR; MC-FR; [Asp^3] MC-FR; MC-WR; [Asp^3] MC-WR; MC-RR; MC-RA	7	Leu<br>Phe<br>Trp<br>Arg	Arg<br>Ala	Position 3 × 2	16	[20]
Microcystis aeruginosa UAM1303	MC-LR; [Asp^3] MC-LR; [MeSer^7] MC-LR; MC-HilR; MC LY; MC-LF; MC-LW	7	Leu<br>Hil	Arg<br>Tyr<br>Phe<br>Trp	Position 3 × 2<br>Position 7 × 2	32	[21]
Microcystis aeruginosa UTEX2666	MC-LA; [Asp^3] MC-LA; MC-LAba; [Asp^3, Glu(OMe)^6] MC-LAba; MC-LR; [Asp^3] MC-LR; didmMC-LR	7	Leu	Ala<br>Aba<br>Arg	Position 3 × 2<br>Position 6 × 2	24	[21]
Microcystis aeruginosa UTEX2670	MC-YA; MC-YL; MC-YM; MC-YM(O); Unidentified MC	5	Tyr	Ala<br>Leu<br>Met<br>Met(O)		-	[21]
Microcystis aeruginosa UV-006	MC-LA; MC-LAba; MC-LV; MC-LL; MC-LR; [Asp^3] MC-LR; Unidentified MCs × 2	8	Leu	Ala<br>Aba<br>Val<br>Leu<br>Arg	Position 3 × 2	≥10	[22]
Microcystis novacekii UAM250	MC-LR; MC-YR; MC-RR	3	Leu<br>Tyr<br>Arg	Arg		3	[23]

Table S1. *Cont.*

Microcystis viridis NIES102	[Asp^3] MC-LR; [Dha^7] MC-LR; [Ser^1, Asp^3, Dha^7] MC-LR; MC-HilR; MC-FR; MC-YR; [Asp^3] MC-YR; MC-HtyR; MC-WR; [Asp^3] MC WR; MC-RR; [Asp^3] MC-RR; Unidentified MCs × 35	47	Leu<br>Hil<br>Phe<br>Tyr<br>Hty<br>Trp<br>Arg	Arg	Position 1 × 2<br>Position 3 × 2<br>Position 7 × 2	-	[1]
Nostoc 152	[Asp^3, ADMAdda^5] MC-VR; [DMAdda^5] MC-LR; [ADMAdda^5] MC-LR; [Mdhb^7] MC-LR; [Ser^1, ADMAdda^5] MC-LR; [Ser^1, Asp^3, ADMAdda^5] MC LR; [Asp^3, DMAdda^5] MC-LR; [ADMAdda^5, MeSer^7] MC-LR; [ADMAdda^5, Dha^7] MC-LR; [Asp^3, ADMAdda^5, Dha^7] MC-LR; [ADMAdda^5] MC-LHar; [DMAdda^5] MC-LHar; [Asp^3, ADMAdda^5] MC-LHar; [ADMAdda^5] MC-HilR; [ADMAdda^5] MC-HilHar; [Asp^3, ADMAdda^5] MC-HilR; Unidentified MCs × 9	25	Val<br>Leu<br>Hil	Arg<br>Har	Position 1 × 2<br>Position 3 × 2<br>Position 5 × 3<br>Position 7 × 3	≥216	[1,24–26]
Nostoc IO-102-I	[ADMAdda^5] MC-LR; [DMAdda^5] MC-LR; [Asp^3, ADMAdda^5] MC LR; [DMAdda^5] MC-HilR; [ADMAdda^5] MC-YR; Unidentified MCs × 15	20	Leu<br>Hil<br>Tyr	Arg	Position 3 × 2<br>Position 5 × 2	-	[1,27]
Nostoc species	[Asp^3, ADMAdda^5, Dhb^7] MC-LR; [Asp^3, ADMAdda^5, Dhb^7] MC HtyR; [Asp^3, ADMAdda^5, Dhb^7] MC-RR	3	Leu<br>Hty<br>Arg	Arg	Position 3 × 1<br>Position 5 × 1<br>Position 7 × 1	3	[28]
Planktothrix Max06	[Asp^3, DMAdda^5] MC-HtyR; [Asp^3] MC-YR; [Asp^3, MeSer^7] MC-HtyR; [Asp^3, MeSer^7] MC-LR; [Asp^3] MC-HtyR; [Asp^3] MC-LR; [Asp^3, Dha^7] MC-LR; [Asp^3] MC-HilR; [Asp^3, Glu(OMe)^6] MC-HtyR; [Asp^3] MC-HphR; [Asp^3, Glu(OMe)^6] MC-LR	11	Leu<br>Hil<br>Tyr<br>Hty<br>Hph	Arg	Position 3 × 1<br>Position 5 × 2<br>Position 6 × 2<br>Position 7 × 3	60	[29]

Table S1. *Cont.*

Planktothrix agardhii	[Asp^3] MC-LR; [Asp^3] MC-RR	2	Leu<br>Arg	Arg	Position 3 ×1	2	[30]
Planktothrix agardhii 213	[Asp^3] MC-LR; [Asp^3, Dha^7] MC-LR; [Asp^3] MC-RR	3	Leu<br>Arg	Arg	Position 3 ×1<br>Position 7 ×2	4	[1]
Planktothrix agardhii CYA 56/3	[Asp^3] MC-LY; [Asp^3] MC-LR; [Asp^3] MC-HtyR; [Asp^3] MC-RR; [Asp^3, Dha^7] MC-RR; [Asp^3] MC-RY; [Asp^3, Dha^7] MC-RY; [Asp^3, DMAdda^5] MC-RY; Unidentified MC	9	Leu<br>Hty<br>Arg	Tyr<br>Arg	Position 3 ×1<br>Position 5 ×2<br>Position 7 ×2	≥24	[31]
Planktothrix agardhii CYA 137	[Asp^3] MC-LY; [Asp^3] MC-LR; [Asp^3] MC-HtyR; [Asp^3] MC-RR; [Asp^3, Dha^7] MC-RR; [Asp^3] MC-RY; [Asp^3, Dha^7] MC-RY; [Asp^3, DMAdda^5] MC-RY; Unidentified MC	9	Leu<br>Hty<br>Arg	Tyr<br>Arg	Position 3 ×1<br>Position 5 ×2<br>Position 7 ×2	≥24	[31]
Planktothrix agardhii CYA 532	[Asp^3] MC-LY; [Asp^3] MC-LR; [Asp^3] MC-HtyR; [Asp^3] MC-RR; [Asp^3, Dha^7] MC-RR; [Asp^3] MC-RY; [Asp^3, Dha^7] MC-RY; [Asp^3, DMAdda^5] MC-RY; Unidentified MC	9	Leu<br>Hty<br>Arg	Tyr<br>Arg	Position 3 ×1<br>Position 5 ×2<br>Position 7 ×2	≥24	[31]
Planktothrix agardhii CYA 537	[Asp^3] MC-LR; [Asp^3] MC-RR; [Asp^3, Dha^7] MC-RR; Unidentified MC	4	Leu<br>Arg	Arg	Position 3 ×1<br>Position 7 ×2	≥4	[31]
Planktothrix agardhii CYA 544	[Asp^3] MC-LR; [Asp^3] MC-RR; [Asp^3, Dha^7] MC-RR; Unidentified MC	4	Leu<br>Arg	Arg	Position 3 ×1<br>Position 7 ×2	≥4	[31]
Planktothrix agardhii NIVA 126/8	[Asp^3] MC-LR; [Asp^3] MC-RR; Unidentified MC	3	Leu<br>Arg	Arg	Position 3 ×1	-	[1]
Planktothrix agardhii PH-123	[Asp^3] MC-LR; [Asp^3, ADMAdda^5] MC-LR; [Asp^3] MC-HtyR; [Asp^3, ADMAdda^5] MC-HtyR	4	Leu<br>Hty	Arg	Position 3 ×1<br>Position 5 ×2	4	[32]
Planktothrix rubescens CYA 406	[Asp^3, Dhb^7] MC-LR; [Asp^3, Dhb^7] MC-HtyR; [Asp^3, Dhb^7] MC-RR; [Asp^3, Dha^7] MC-RR; Unidentified MC	5	Leu<br>Hty<br>Arg	Arg	Position 3 ×1<br>Position 7 ×2	≥6	[31]

Table S1. *Cont.*

Planktothrix rubescens CYA 408	[Asp^3, Dhb^7] MC-LR; [Asp^3, Dhb^7] MC-HtyR; [Asp^3, Dhb^7] MC-RR; [Asp^3, Dha^7] MC-RR; Unidentified MC	5	Leu<br>Hty<br>Arg	Arg	Position 3 × 1<br>Position 7 × 2	≥6	[31]
Planktothrix rubescens No80	[Asp^3, Dhb^7] MC-LY; [Asp^3, Dhb^7] MC-LW; [Asp^3, Dhb^7] MC-HtyY; [Asp^3, Dhb^7] MC-HtyHty; [Asp^3, Dhb^7] MC-HtyW	5	Leu<br>Hty	Tyr<br>Hty<br>Trp	Position 3 × 1<br>Position 7 × 1	6	[33,34]

[a] An assessment of the microcystin diversity of 49 microcystin-producing strains reported in scientific journals; [b] Number of microcystins observed, including unidentified microcystins which the researchers noted during the studies; [c] Amino acids incorporated into position two of the microcystins reported to be produced by the cyanobacterial strain; [d] Amino acids incorporated into position four of the microcystins reported to be produced by the cyanobacterial strain; [e] Potential number of microcystins which could be produced by the cyanobacterial strain according to the information collected; In some cases this is omitted as the presence of unidentified microcystins makes this value difficult to estimate.

Table S2. Tandem mass spectrometry fragment assignments for the CAWBG11 -RR microcystin (MC) congeners observed by electrospray ionization collision-induced dissociation.

Fragment Assignment [a]	MC-RR		[Asp³] MC-RR	
	$[M + 2H]^{2+}$	$[M + H]^{+}$	$[M + 2H]^{2+}$	$[M + H]^{+}$
M	519.8	1038.6	512.8	1024.6
M − H_2O	510.7	1020.4 [b]	503.7	1006.4 [b]
M − Mdha − H_2O	469.2	937.4 [b]	462.2	923.4 [b]
M − Adda sidechain	452.8	904.4	445.7	890.3
M − Adda sidechain − H_2O	443.7	886.4	436.6	872.3
M − Adda	363.2	725.3	356.1	711.3
M − Adda − H_2O	354.2	707.3	347.1	693.3
Arg-Adda-Glu − NH_3		582.2		582.2
Arg-Adda − NH_3 + H		453.2		453.2
Arg-Adda-Glu − CO		571.3		571.2
(Me)Asp-Arg-Adda-Glu		728.3		714.2
(Me)Asp-Arg-Adda		599.3		585.2
Arg-Adda-Glu		599.3		599.2
Mdha-Ala-Arg-(Me)Asp-Arg	298.2	596.3	291.2	582.2
Mdha-Ala-Arg-(Me)Asp		440.2		426.1
Mdha-Ala-Arg		311.2		311.1
Mdha-Ala		155.1		155.0
Adda′-Glu-Mdha		375.2		375.1
Adda′		163.1		163.0
(Me)Asp-Arg		286.2		272.1
Arg		157.1		157.1

[a] Adda′ = Adda minus NH_2 and the sidechain ($C_9H_{11}O$); [b] $[M + H]^{+}$ ion was deconvoluted from the $[M + 2H]^{2+}$ ion.

Table S3. Tandem mass spectrometry fragment assignments for the CAWBG11 -XR microcystin (MC) congeners observed by matrix-assisted laser desorption/ionization post-source decay and electrospray ionization collision-induced dissociation.

**Fragment Assignment** [a]	**MC-LR** *X* **= 113 Da**	**[Asp³] MC-LR** *X* **= 113 Da**	**MC-FR** *X* **= 147 Da**	**[Asp³] MC-FR** *X* **= 147 Da**	**MC-YR** *X* **= 163 Da**	**MC-WR** *X* **= 186 Da**	**[Asp³] MC-WR** *X* **= 186 Da**
M + H	995	981	1029	1015	1045	1068	1054
M − Ala + H	924	910	958	944	974	997	
M − $CH_2NHCN_2H_3$ + H	923	909	957	943	973	996	
M − (Me)Asp + H	866	866	900	900	916	939	939
M − Glu + H	866	852	900	886	916	939	925
M − Adda sidechain + H	861	847	895	881	911	934	920
(Me)Asp-Arg-Adda-Glu + H	728	714	728	714	728	728	714
(Me)Asp-Arg-Adda + H	599	585	599	585	599	599	585
Arg-Adda-Glu + H	599	599	599	599	599	599	599
Arg-Adda + H	470	470	470	470	470	470	470
Mdha-Ala-*X*-(Me)Asp-Arg + NH_4	570	556	604	590	620	643	629
Ala-*X*-(Me)Asp-Arg + NH_4	487	473	521	507	537	560	546
Mdha-Ala-*X*-(Me)Asp-Arg + H	553	539	587	573	603	626	612
Ala-*X*-(Me)Asp-Arg + H	470	456	504	490	520	543	529
X-(Me)Asp-Arg + H	399		433	419	449		
Mdha-Ala-*X*-(Me)Asp + H	397	383	431		447	470	456
Mdha-Ala-*X* + H	268	268	302	302	318	341	341
Mdha-Ala + H	155	155	155	155	155	155	155
Adda′-Glu-Mdha-Ala + H	446	446	446	446	446	446	446
Adda′-Glu-Mdha + H	375	375	375	375	375	375	375
Adda′ + H	163	163	163	163	163	163	163
Glu-Mdha + H	213	213	213	213	213	213	213
Adda sidechain	135	135	135	135	135	135	135
Arg related ions	70/84/112/174	70/84/112/174	70/84/112/174	70/84/112/174	70/84/112/174	70/84/112/174	70/84/112/174
X immonium	86	86	120	120	136	159	159

[a] *X* = Position two amino acid; Adda′ = Adda minus NH_2 and the sidechain ($C_9H_{11}O$); $CH_2NHCN_2H_3$ is a fragment of the arginine sidechain; Fragment ions containing NH_3 and CO losses have been omitted.

Table S4. Tandem mass spectrometry fragment assignments for the CAWBG11 -RZ microcystin (MC) congeners observed by matrix-assisted laser desorption/ionization post-source decay and electrospray ionization collision-induced dissociation.

**Fragment Assignment** [a]	**MC-RA** *Z* = 71 Da	**[Asp³] MC-RA** *Z* = 71 Da	**MC-RAba** *Z* = 85 Da	**[Asp³] MC-RAba** *Z* = 85 Da	**MC-RL** *Z* = 113 Da
M + H	953	939	967	953	995
M − H_2O + H	935	921	949	935	977
M − COOH + H	908	894	922	908	950
M − Z + H	882	868	882	868	882
M − $CH_2NHCN_2H_3$ + H	881	867	895	881	923
M − Glu + H	824	810	838	824	866
M − (Me)Asp + H	824	824	838	838	866
M − Adda sidechain + H	819	787	833	819	861
Mdha-Ala-Arg-(Me)Asp-Z + NH_4	528	514	542	528	570
Mdha-Ala-Arg-(Me)Asp-Z − H_2O + NH_4	510	496	524	510	552
Mdha-Ala-Arg-(Me)Asp-Z + H	511	497	525	511	553
Mdha-Ala-Arg-(Me)Asp − $CH_2NHCN_2H_3$ + H	368	354	368	354	368
Mdha-Ala-Arg-(Me)Asp + H	440	426	440	426	440
Mdha-Ala-Arg + H	311	311	311	311	311
Mdha-Ala + H	155	155	155		
Arg-(Me)Asp-Z + H	357	343		357	
Glu-Mdha-Ala-Arg − COOH + H	395	395	395	395	395
Glu-Mdha-Ala-Arg − $CH_2NHCN_2H_3$ + H	368	368	368	368	368
Glu-Mdha-Ala-Arg + H	440	440	440	440	440
Glu-Mdha + H	213		213		
Adda′-Glu-Mdha + H	375	375	375	375	375
Adda′ + H	163	163	163		

[a] *Z* = Position four amino acid; Adda′ = Adda minus NH_2 and the sidechain ($C_9H_{11}O$); $CH_2NHCN_2H_3$ is a fragment of the arginine sidechain.

Table S5. Tandem mass spectrometry fragment assignments for the CAWBG11 -XA microcystin (MC) congeners observed by electrospray ionization collision-induced dissociation.

Fragment Assignment [a]	MC-LA<br>*X* = 113 Da	[Asp[3]] MC-LA<br>*X* = 113 Da	MC-FA<br>*X* = 147 Da	[Asp[3]] MC-FA<br>*X* = 147 Da	MC-YA<br>*X* = 163 Da	MC-WA<br>*X* = 186 Da	[Asp[3]] MC-WA<br>*X* = 186 Da
M + H	910	896	944	930	960	983	969
M − H_2O + H	892	878	926	912	942	965	951
M − Mdha − H_2O + H	809	795	843	829		882	868
M − Adda sidechain + H	776	762	810	796	826	849	835
M − Adda sidechain − H_2O + H	758	744	792	778	808	831	817
M − Adda + H	597	583	631	617	647	670	656
M − Adda − H_2O + H	579	565	613	599	629	652	
Adda-Glu-Mdha-Ala-*X*-(Me)Asp − NH_3 + H	822	808	856	842	872	895	881
Adda-Glu-Mdha-Ala-*X* − NH_3 + H	693	693	727	727	743	766	766
Adda-Glu-Mdha-Ala − NH_3 + H	580	580	580	580	580	580	580
Adda-Glu-Mdha − NH_3 + H	509	509	509	509	509	509	509
Adda′-Glu-Mdha-Ala-*X* + H	559	559	593	593	609	632	632
Adda′-Glu-Mdha-Ala + H	446	446	446	446	446	446	446
Adda′-Glu-Mdha + H	375	375	375	375	375	375	375
Mdha-Ala-*X*-(Me)Asp-Ala + NH_4	485	471	519	505	535	558	
Ala-*X*-(Me)Asp-Ala + NH_4	402	388	436	422	452	475	461
X-(Me)Asp-Ala + NH_4	331	317	365	351	381	404	390
Mdha-Ala-*X*-(Me)Asp-Ala + H	468	454	502	334	518	541	527
Ala-*X*-(Me)Asp-Ala + H	385	371	419	405	435	458	444
X-(Me)Asp-Ala + H	314	300	348	488	364	387	373

[a] *X* = Position two amino acid; Adda′ = Adda minus NH_2 and the sidechain ($C_9H_{11}O$).

Table S6. Tandem mass spectrometry fragment assignments for the CAWBG11 -XAba microcystin (MC) congeners observed by electrospray ionization collision-induced dissociation.

**Fragment Assignment** [a]	**MC-LAba** ***X*** **= 113 Da**	**MC-FAba** ***X*** **= 147 Da**	**MC-WAba** ***X*** **= 186 Da**
M + H	924	958	997
M − H_2O + H	906	940	979
M − Mdha − H_2O + H	823	857	896
M − Adda sidechain + H	790	824	863
M − Adda sidechain − H_2O + H	772	806	845
M − Adda + H	611	645	684
M − Adda − H_2O + H	593	627	666
Adda-Glu-Mdha-Ala-*X*-Masp − NH_3 + H		856	895
Adda-Glu-Mdha-Ala-*X* − NH_3 + H	693	727	766
Adda-Glu-Mdha-Ala − NH_3 + H	580	580	580
Adda-Glu-Mdha − NH_3 + H	509	509	509
Adda′-Glu-Mdha-Ala-*X* + H	559	593	632
Adda′-Glu-Mdha-Ala + H	446	446	446
Adda′-Glu-Mdha + H	375	375	375
Mdha-Ala-*X*-Masp-Aba + NH_4	499	533	572
Ala-*X*-Masp-Aba + NH_4	416	450	489
X-Masp-Aba + NH_4	345	379	418
Mdha-Ala-*X*-Masp-Aba + H	482	516	555
Ala-*X*-Masp-Aba + H	399	433	472
X-Masp-Aba + H	328	362	401

[a] *X* = Position two amino acid; Adda′ = Adda minus NH_2 and the sidechain ($C_9H_{11}O$).

Table S7. Tandem mass spectrometry fragment assignments for the CAWBG11 -XL microcystin (MC) congeners observed by electrospray ionization collision-induced dissociation.

**Fragment Assignment** [a]	**MC-LL** ***X*** **= 113 Da**	**MC-FL** ***X*** **= 147 Da**	**MC-WL** ***X*** **= 186 Da**
M + H	952	986	1025
M − NH_3 + H	935	969	1008
M − H_2O + H	934	968	1007
M − Mdha − H_2O + H	851	885	924
M − Adda sidechain + H	818	852	891
M − Adda sidechain − H_2O + H	800	834	873
M − Adda + H	639	673	712
M − Adda − H_2O + H	621	655	694
Adda-Glu-Mdha-Ala-*X*-Masp − NH_3 + H		856	895
Adda-Glu-Mdha-Ala-*X* − NH_3 + H	693	727	766
Adda-Glu-Mdha-Ala − NH_3 + H	580	580	580
Adda-Glu-Mdha − NH_3 + H	509	509	509
Glu-Mdha-Ala-*X* + H	397	431	470
Adda′-Glu-Mdha-Ala-*X* + H	559	593	632
Adda′-Glu-Mdha-Ala + H	446	446	446
Adda′-Glu-Mdha + H	375	375	375
Mdha-Ala-*X*-Masp-Leu + NH_4		561	600
Ala-*X*-Masp-Leu + NH_4		478	517
X-Masp-Leu + NH_4		407	446
Unidentified fragment ion	440	474	513
Unidentified fragment ion	535	535	535
Mdha-Ala-*X*-Masp-Leu + H	509	544	583
Ala-*X*-Masp-Leu + H	426	461	500
X-Masp-Leu + H	355	390	429

[a] *X* = Position two amino acid; Adda′ = Adda minus NH_2 and the sidechain ($C_9H_{11}O$).

Table S8. Electrospray high-resolution mass spectrometry data for microcystins (MC) present in sufficient quantities in *Microcystis* CAWBG11.

**Microcystin**	**Measured *m/z***		**Proposed Formula**	**Expected *m/z***	**Deviation**
MC-LR (**1**)	995.5560	$[M + H]^+$	$C_{49}H_{75}N_{10}O_{12}$	995.5560	+0.1 ppm
MC-RR (**3**)	519.7884	$[M + 2H]^{2+}$	$C_{49}H_{77}N_{13}O_{12}$	519.7902	−3.4 ppm
MC-YR (**4**)	1045.5364	$[M + H]^+$	$C_{52}H_{73}N_{10}O_{13}$	1045.5353	+1.1 ppm
[Asp^3] MC-LR (**5**)	981.5369	$[M + H]^+$	$C_{48}H_{73}N_{10}O_{12}$	981.5404	−3.6 ppm
[Asp^3] MC-FR (**6**)	1015.5207	$[M + H]^+$	$C_{51}H_{71}N_{10}O_{12}$	1015.5247	−4.0 ppm
MC-FR (**7**)	1029.5411	$[M + H]^+$	$C_{52}H_{72}N_{10}O_{12}$	1029.5404	+0.6 ppm
[Asp^3] MC-WR (**8**)	1054.5398	$[M + H]^+$	$C_{53}H_{72}N_{11}O_{12}$	1054.5356	+3.9 ppm
MC-WR (**9**)	1068.5465	$[M + H]^+$	$C_{54}H_{74}N_{11}O_{12}$	1068.5513	−4.5 ppm
MC-RA (**11**)	953.5122	$[M + H]^+$	$C_{46}H_{69}N_{10}O_{12}$	953.5091	+3.3 ppm
MC-RAba (**13**)	967.5259	$[M + H]^+$	$C_{47}H_{71}N_{10}O_{12}$	967.5247	+1.1 ppm
[Asp^3] MC-LA (**16**)	918.4592	$[M + Na]^+$	$C_{45}H_{67}N_7O_{12}Na$	918.4583	+1.0 ppm
MC-LA (**17**)	910.4936	$[M + H]^+$	$C_{46}H_{68}N_7O_{12}$	910.4920	+1.7 ppm
MC-FA (**19**)	966.4550	$[M + Na]^+$	$C_{49}H_{65}N_7O_{12}Na$	966.4583	−3.3 ppm
MC-WA (**21**)	1005.4650	$[M + Na]^+$	$C_{51}H_{66}N_8O_{12}Na$	1005.4692	−4.3 ppm
MC-LAba (**22**)	946.4912	$[M + Na]^+$	$C_{47}H_{69}N_7O_{12}Na$	946.4896	+1.7 ppm
MC-FAba (**23**)	980.4744	$[M + Na]^+$	$C_{50}H_{67}N_7O_{12}Na$	980.4740	+0.4 ppm
MC-WAba (**24**)	1019.4836	$[M + Na]^+$	$C_{52}H_{68}N_8O_{12}Na$	1019.4849	−1.3 ppm

Figure S1. Advanced Marfey's amino acid analysis of MC-RA; extracted ion chromatograms of hydrolyzed MC-RA derivatized with L-FDLA.

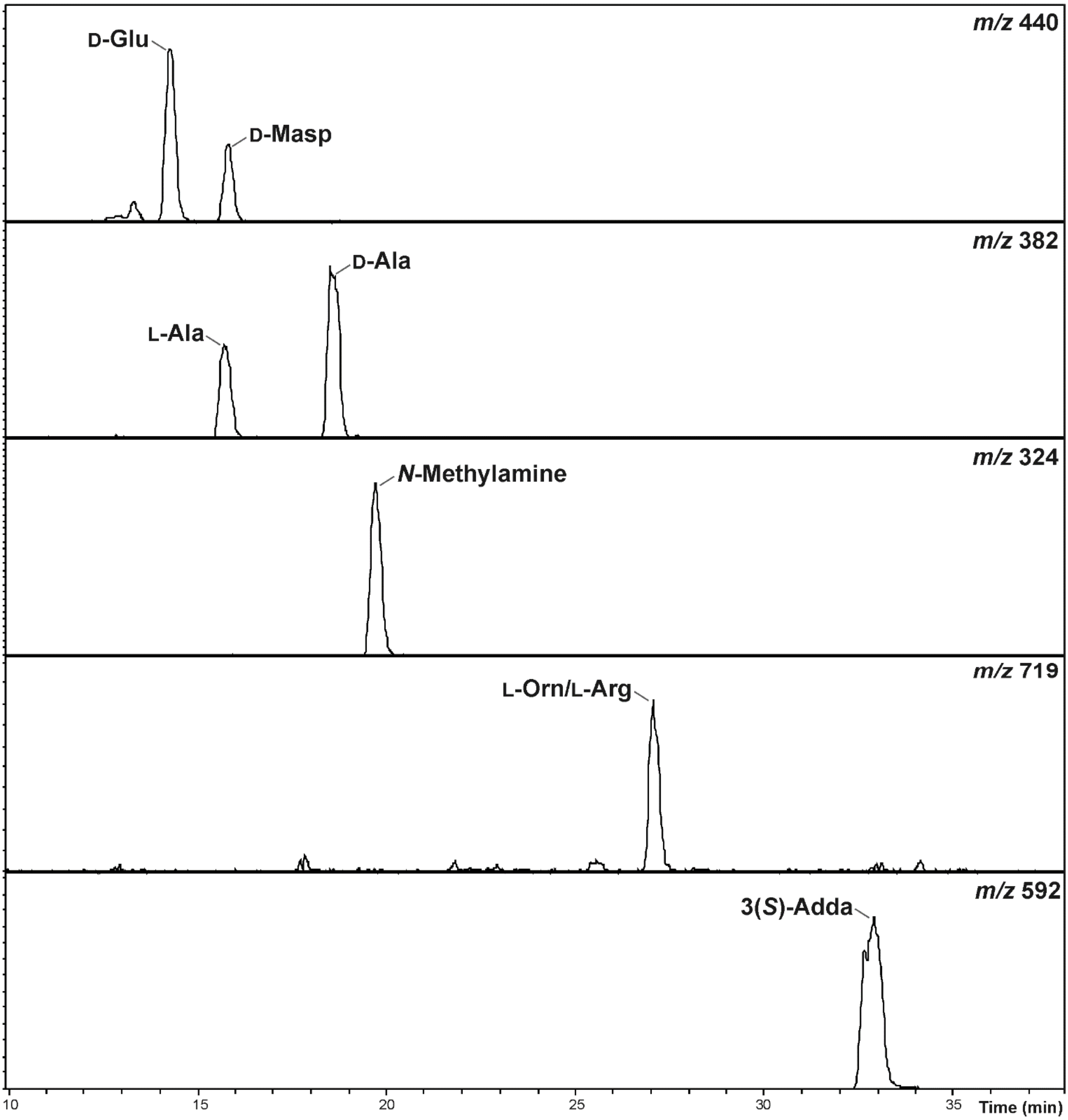

Figure S2. Advanced Marfey's amino acid analysis of MC-RAba; extracted ion chromatograms of hydrolyzed MC-RAba derivatized with L-FDLA.

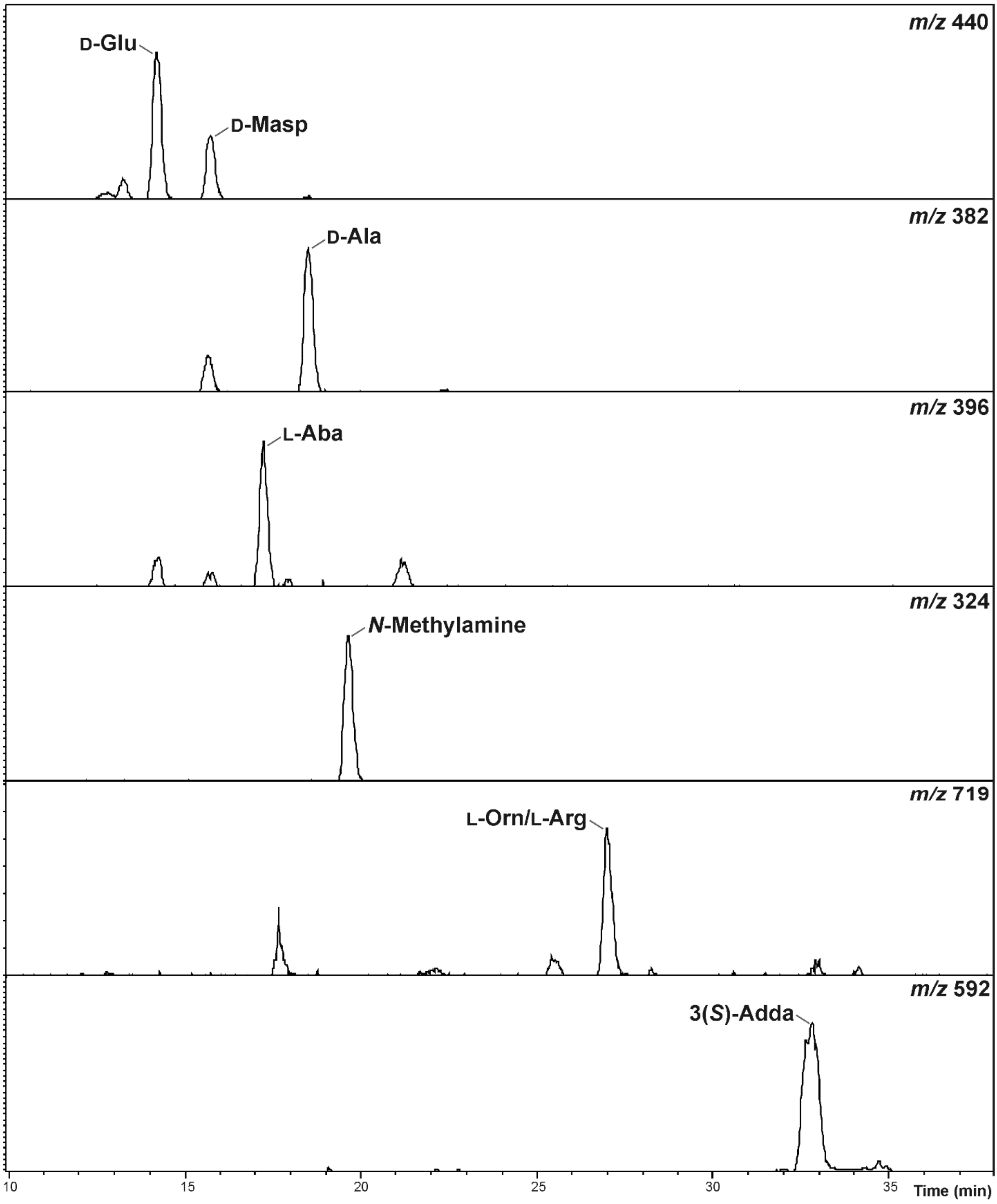

Figure S3. Box plots representing the spread in the number of microcystin congeners produced by reported cyanobacterial strains. Plots depict the number of microcystin congeners identified; 49 strains (**a**); the number of microcystin congeners observed; 49 strains (**b**) and the potential number of congeners which could be produced according to the reported data; 33 strains (**c**).

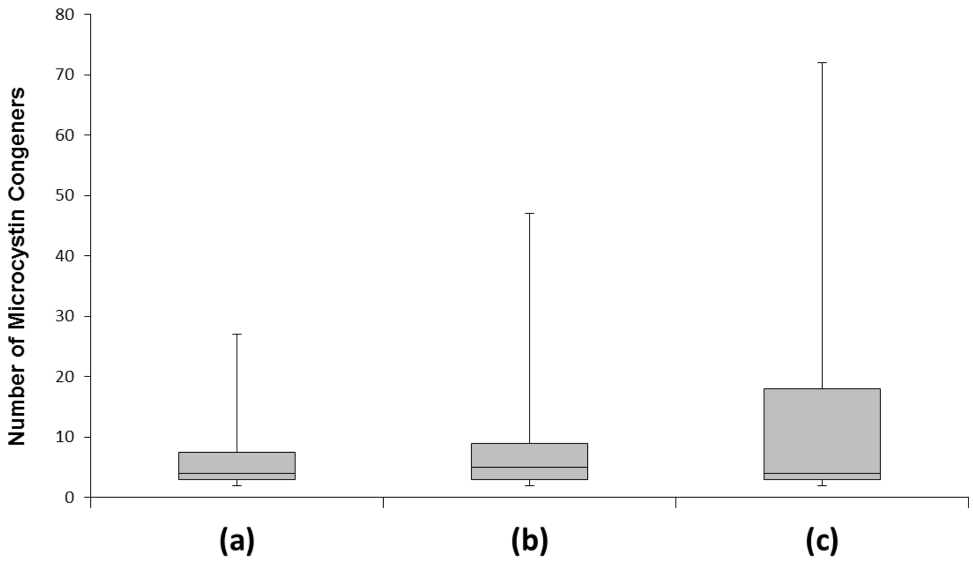

Figure S4. Microscopic images of *Microcystis* CAWBG11 acquired on an Olympus IX70 inverted microscope at 100 × magnification (**a**) and at 1000 × magnification (**b**).

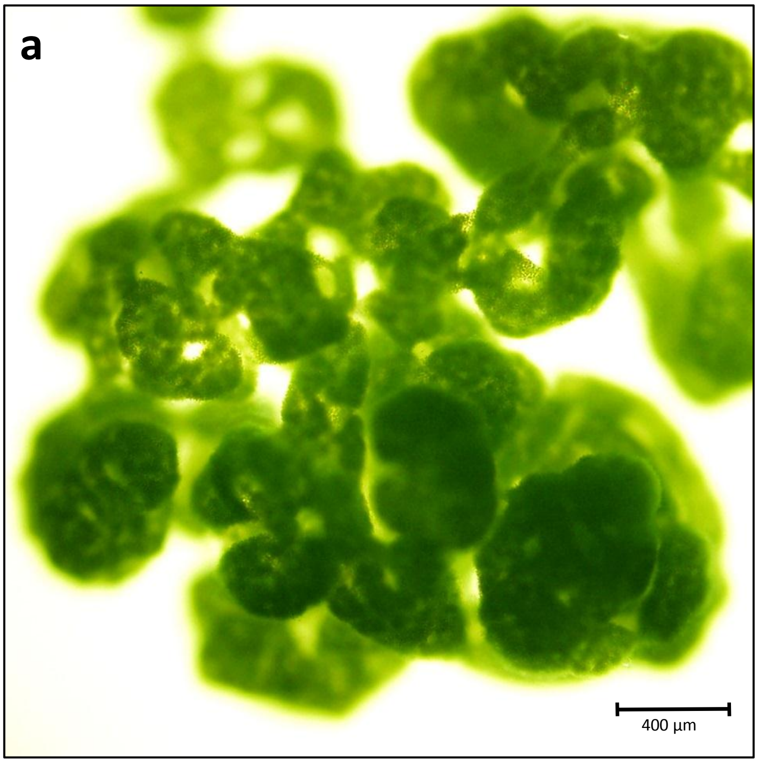

Figure S4. *Cont.*

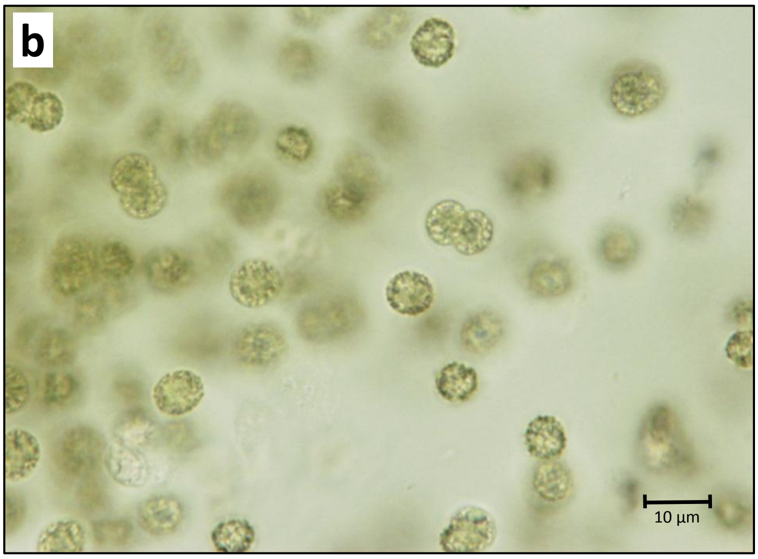

References

1. Fewer, D.; Rouhiainen, L.; Jokela, J.; Wahlsten, M.; Laakso, K.; Wang, H.; Sivonen, K. Recurrent adenylation domain replacement in the microcystin synthetase gene cluster. *BMC Evol. Biol.* **2007**, *7*, 1–11.
2. Sivonen, K.; Namikoshi, M.; Evans, W.R.; Carmichael, W.W.; Sun, F.; Rouhiainen, L.; Luukkainen, R.; Rinehart, K.L. Isolation and characterization of a variety of microcystins from seven strains of the cyanobacterial genus Anabaena. *Appl. Environ. Microbiol.* **1992**, *58*, 2495–2500.
3. Namikoshi, M.; Sivonen, K.; Evans, W.R.; Carmichael, W.W.; Rouhiainen, L.; Luukkainen, R.; Rinehart, K.L. Structures of three new homotyrosine-containing microcystins and a new homophenylalanine variant from *Anabaena* sp. strain 66. *Chem. Res. Toxicol.* **1992**, *5*, 661−666.
4. Rapala, J.; Sivonen, K. Assessment of environmental conditions that favor hepatotoxic and neurotoxic *Anabaena* spp. strains cultured under light limitation at different temperatures. *Microb. Ecol.* **1998**, *36*, 181–192.
5. Fujii, K.; Sivonen, K.; Nakano, T.; Harada, K.-I. Structural elucidation of cyanobacterial peptides encoded by peptide synthetase gene in Anabaena species. *Tetrahedron* **2002**, *58*, 6863–6871.
6. Namikoshi, M.; Yuan, M.; Sivonen, K.; Carmichael, W.W.; Rinehart, K.L.; Rouhiainen, L.; Sun, F.; Brittain, S.; Otsuki, A. Seven new microcystins possessing two L-glutamic acid units, isolated from *Anabaena* sp. strain 186. *Chem. Res. Toxicol.* **1998**, *11*, 143–149.
7. Namikoshi, M.; Sivonen, K.; Evans, W.R.; Carmichael, W.W.; Sun, F.; Rouhiainen, L.; Luukkainen, R.; Rinehart, K.L. Two new L-serine variants of microcystins-LR and -RR from *Anabaena* sp. strains 202 A1 and 202 A2. *Toxicon* **1992**, *30*, 1457–1464.
8. Sivonen, K.; Skulberg, O.M.; Namikoshi, M.; Evans, W.R.; Carmichael, W.W.; Rinehart, K.L. Two methyl ester derivatives of microcystins, cyclic heptapeptide hepatotoxins, isolated from *Anabaena flos-aquae* strain CYA 83/1. *Toxicon* **1992**, *30*, 1465–1471.

9. Harada, K.-I.; Ogawa, K.; Kimura, Y.; Murata, H.; Suzuki, M.; Thorn, P.M.; Evans, W.R.; Carmichael, W.W. Microcystins from *Anabaena flos-aquae* NRC 525−17. *Chem. Res. Toxicol.* **1991**, *4*, 535–540.
10. Fiore, M.F.; Genuário, D.B.; da Silva, C.S.P.; Shishido, T.K.; Moraes, L.A.B.; Neto, R.C.; Silva-Stenico, M.E. Microcystin production by a freshwater spring cyanobacterium of the genus Fischerella. *Toxicon* **2009**, *53*, 754–761.
11. Fastner, J.; Erhard, M.; von Döhren, H. Determination of oligopeptide diversity within a natural population of *Microcystis* spp. (Cyanobacteria) by typing single colonies by matrix-assisted laser desorption ionization-time of flight mass spectrometry. *Appl. Environ. Microbiol.* **2001**, *67*, 5069–5076.
12. Beresovsky, D.; Hadas, O.; Livne, A.; Sukenik, A.; Kaplan, A.; Carmeli, S. Toxins and biologically active secondary metabolites of *Microcystis* sp. isolated from Lake Kinneret. *Isr. J. Chem.* **2006**, *46*, 79–87.
13. Tonk, L.; Welker, M.; Huisman, J.; Visser, P.M. Production of cyanopeptolins, anabaenopeptins, and microcystins by the harmful cyanobacteria *Anabaena* 90 and *Microcystis* PCC 7806. *Harmful Algae* **2009**, *8*, 219–224.
14. Dai, R.; Liu, H.; Qu, J.; Zhao, X.; Hou, Y. Effects of amino acids on microcystin production of the *Microcystis aeruginosa*. *J. Hazard. Mater.* **2009**, *161*, 730–736.
15. Diehnelt, C.W.; Dugan, N.R.; Peterman, S.M.; Budde, W.L. Identification of microcystin toxins from a strain of *Microcystis aeruginosa* by liquid chromatography introduction into a hybrid linear ion trap-fourier transform ion cyclotron resonance mass spectrometer. *Anal. Chem.* **2006**, *78*, 501–512.
16. Sivonen, K.; Namikoshi, M.; Evans, W.R.; Gromov, B.V.; Carmichael, W.W.; Rinehart, K.L. Isolation and structures of five microcystins from a Russian *Microcystis aeruginosa* strain CALU 972. *Toxicon* **1992**, *30*, 1481–1485.
17. Mazur-Marzec, H.; Browarczyk-Matusiak, G.; Forycka, K.; Kobos, J.; Plinski, M. Morphological, genetic, chemical and ecophysiological characterisation of two *Microcystis aeruginosa* isolates from the Vistula Lagoon, southern Baltic. *Oceanologia* **2010**, *52*, 127–146.
18. Erhard, M.; von Döhren, H.; Jungblut, P.R. Rapid typing and elucidation of new secondary metabolites of intact cyanobacteria using MALDI-TOF mass spectrometry. *Nat. Biotechnol.* **1997**, *15*, 906–909.
19. Robillot, C.; Vinh, J.; Puiseux-Dao, S.; Hennion, M.-C. Hepatotoxin production kinetics of the cyanobacterium *Microcystis aeruginosa* PCC 7820, as determined by HPLC-mass spectrometry and protein phosphatase bioassay. *Environ. Sci. Technol.* **2000**, *34*, 3372–3378.
20. Lee, T.-H.; Chou, H.-N. Isolation and identification of seven microcystins from a cultured M.TN-2 strain of *Microcystis aeruginosa*. *Bot. Bull. Academ. Sin.* **2000**, *41*, 197–202.
21. Del Campo, F.F.; Ouahid, Y. Identification of microcystins from three collection strains of Microcystis aeruginosa. *Environ. Pollut.* **2010**, *158*, 2906–2914.
22. Gademann, K.; Portmann, C.; Blom, J.F.; Zeder, M.; Jüttner, F. Multiple toxin production in the cyanobacterium *Microcystis*: Isolation of the toxic protease inhibitor cyanopeptolin 1020. *J. Nat. Prod.* **2010**, *73*, 980–984.

23. Li, H.; Murphy, T.; Guo, J.; Parr, T.; Nalewajko, C. Iron-stimulated growth and microcystin production of *Microcystis novacekii* UAM 250. *Limnol.-Ecol. Manag. Inland Waters* **2009**, *39*, 255–259.
24. Namikoshi, M.; Rinehart, K.L.; Sakai, R.; Sivonen, K.; Carmichael, W.W. Structures of three new cyclic heptapeptide hepatotoxins produced by the cyanobacterium (blue-green alga) *Nostoc* sp. strain 152. *J. Org. Chem.* **1990**, *55*, 6135–6139.
25. Sivonen, K.; Carmichael, W.W.; Namikoshi, M.; Rinehart, K.L.; Dahlem, A.M.; Niemela, S.I. Isolation and characterization of hepatotoxic microcystin homologs from the filamentous freshwater cyanobacterium *Nostoc* sp. strain 152. *Appl. Environ. Microbiol.* **1990**, *56*, 2650–2657.
26. Sivonen, K.; Namikoshi, M.; Evans, W.R.; Fardig, M.; Carmichael, W.W.; Rinehart, K.L. Three new microcystins, cyclic heptapeptide hepatotoxins, from *Nostoc* sp. strain 152. *Chem. Res. Toxicol.* **1992**, *5*, 464–469.
27. Oksanen, I.; Jokela, J.; Fewer, D.P.; Wahlsten, M.; Rikkinen, J.; Sivonen, K. Discovery of rare and highly toxic microcystins from lichen-associated cyanobacterium *Nostoc* sp. strain IO-102-I. *Appl. Environ. Microbiol.* **2004**, *70*, 5756–5763.
28. Beattie, K.A.; Kaya, K.; Sano, T.; Codd, G.A. Three dehydrobutyrine-containing microcystins from Nostoc. *Phytochemistry* **1998**, *47*, 1289–1292.
29. Welker, M.; Christiansen, G.; von Döhren, H. Diversity of coexisting Planktothrix (Cyanobacteria) chemotypes deduced by mass spectral analysis of microystins and other oligopeptides. *Arch. Microbiol.* **2004**, *182*, 288–298.
30. Tonk, L.; Visser, P.M.; Christiansen, G.; Dittmann, E.; Snelder, E.O.F.M.; Wiedner, C.; Mur, L.R.; Huisman, J. The microcystin composition of the cyanobacterium *Planktothrix agardhii* changes toward a more toxic variant with increasing light intensity. *Appl. Environ. Microbiol.* **2005**, *71*, 5177–5181.
31. Miles, C.O.; Sandvik, M.; Haande, S.; Nonga, H.; Ballot, A. First use of LC-MS analysis with thiol derivatization to differentiate [Dhb^7]- from [Mdha^7]-microcystins: Analysis of cyanobacterial blooms, *Planktothrix* cultures and European crayfish from Lake Steinsfjorden, Norway. *Environ. Sci. Technol.* **2013**, *47*, 4080–4087.
32. Laub, J.; Henriksen, P.; Brittain, S.M.; Wang, J.; Carmichael, W.W.; Rinehart, K.L.; Moestrup, Ø. [ADMAdda^5]-microcystins in *Planktothrix agardhii* strain PH-123 (cyanobacteria)-importance for monitoring of microcystins in the environment. *Environ. Toxicol.* **2002**, *17*, 351–357.
33. Christiansen, G.; Yoshida, W.Y.; Blom, J.F.; Portmann, C.; Gademann, K.; Hemscheidt, T.; Kurmayer, R. Isolation and structure determination of two microcystins and sequence comparison of the McyABC adenylation domains in *Planktothrix* species. *J. Nat. Prod.* **2008**, *71*, 1881–1886.
34. Niedermeyer, T.H.J.; Schmieder, P.; Kurmayer, R. Isolation of microcystins from the cyanobacterium *Planktothrix rubescens* strain No80. *Nat. Prod. Bioprospect.* **2014**, *4*, 37–45.

© 2014 by the authors; licensee MDPI, Basel, Switzerland. This article is an open access article distributed under the terms and conditions of the Creative Commons Attribution license (http://creativecommons.org/licenses/by/4.0/).
